# Divergent Effects of *Circoviridae* Capsid Proteins on Type I Interferon Signaling

**DOI:** 10.3390/pathogens14010068

**Published:** 2025-01-13

**Authors:** Anon H. Kosaka, Chen-Yu Huang, Zih-Ying Lu, Hua-Zhen Hsing, Amonrat Choonnasard, Rissar Siringo Ringo, Kuo Pin Chuang, Akatsuki Saito

**Affiliations:** 1 Department of Veterinary Science, Faculty of Agriculture, University of Miyazaki, Miyazaki 889-2192, Japan; gf21011@student.miyazaki-u.ac.jp (A.H.K.); amonrat_choonnasard@med.miyazaki-u.ac.jp (A.C.); rissar_siringo_ringo@med.miyazaki-u.ac.jp (R.S.R.); 2 Graduate Institute of Animal Vaccine Technology, College of Veterinary Medicine, National Pingtung University of Science and Technology, Pingtung 912, Taiwan; 741852963s.si@gmail.com (C.-Y.H.); annie7311@gmail.com (Z.-Y.L.); nina4802090@gmail.com (H.-Z.H.); kpchuang@mail.npust.edu.tw (K.P.C.); 3 Graduate School of Medicine and Veterinary Medicine, University of Miyazaki, Miyazaki 889-1692, Japan; 4 Center for Animal Disease Control, University of Miyazaki, Miyazaki 889-2192, Japan

**Keywords:** *Circoviridae*, capsid proteins, interferon β signaling

## Abstract

Viruses in the *Circoviridae* family can infect mammals and birds. Porcine circovirus type 2 (PCV2) significantly affects the livestock industry by causing porcine circovirus-associated diseases, such as postweaning multisystem wasting syndrome, respiratory disease complex, and dermatitis nephropathy syndrome. Additionally, beak and feather disease virus in parrots, canine circovirus in dogs, and columbid circovirus (pigeon circovirus) in racing pigeons induce immunosuppression, followed by secondary infections in these hosts. Although the PCV2 capsid protein has been demonstrated to inhibit type I interferon (IFN) signaling, the molecular mechanisms of *Circoviridae*-induced immunosuppression are largely unknown. In this study, we examined whether these functions are conserved across *Circoviridae* capsid proteins. Our results illustrated that although the nuclear localization of capsid proteins is conserved, their effects on IFN-β signaling vary by species, revealing the diverse roles of *Circoviridae* capsid proteins in modulating immune responses.

## 1. Introduction

The *Circoviridae* family comprises small icosahedral viruses without an envelope protein and featuring a circular single-stranded DNA genome of 1600–2200 nucleotides. The *Circoviridae* family has two genera, namely *Circovirus* and *Cyclovirus* (Virus Taxonomy: 2023 Release; https://ictv.global/taxonomy, accessed on 13 November 2024). Several viruses in the *Circoviridae* family cause disease in mammals and birds, and in porcine circovirus type 2 (PCV2) particularly has significantly impacted the livestock industry. PCV2 causes several conditions collectively termed porcine circovirus-associated diseases (PCVADs), including postweaning multisystemic wasting syndrome, porcine respiratory disease complex, and porcine dermatitis nephropathy syndrome [1,2,3,4]. Conversely, porcine circovirus type 1 (PCV1), discovered in the porcine kidney-derived PK-15 cell line approximately 50 years ago, is considered nonpathogenic [1]. PCV3 was also identified in the US in 2015 in a case of porcine dermatitis and nephropathy syndrome (PDNS) [5,6], followed by the detection of PCV4 in pigs with signs of PDNS in China in 2019 [7]. Thus, porcine circoviruses are of high economic importance to the livestock industry.

Beak and feather disease virus (BFDV) is an important pathogen in parrots with psittacine beak and feather disease, and it has been present in Australia for at least 10 million years [8]. This virus substantially hampers conservation efforts for endangered parrots, causing feather damage, abnormal beak formation, and immunodeficiency [9]. Canine circovirus (CanineCV) causes severe symptoms in the presence of other pathogens, such as co-infection of CanineCV and canine parvovirus type 2 in puppies [10]. Additionally, columbid circovirus (pigeon circovirus [PiCV]) poses the greatest health and economic damage in young racing pigeons, inducing young pigeon disease syndrome with a secondary infection attributable to immunosuppression [11]. Therefore, immunosuppression is a common pathogenic feature of *Circoviridae*-related infections.

However, the molecular mechanism of immunosuppression following circovirus infection is largely unknown. Previous studies indicated that the PCV2 capsid protein inhibits type I interferon (IFN) signaling [12]. Specifically, it suppresses IFN-β promoter activity driven by stimulator of interferon genes, TANK-binding kinase 1, and interferon regulatory factor 3 (IRF3) [13], and it reduces phosphorylated IRF3 protein levels in the nucleus [14,15,16]. Supporting the mechanism of action, PCV2 capsid protein localizes in the nucleus [17,18]. However, whether these phenotypes are PCV2-specific remains unclear.

In this study, we investigated whether these functions are conserved across *Circoviridae* family capsid proteins. Our results demonstrated that although nuclear localization of the capsid protein is conserved among the *Circoviridae* family, the effect on IFN-β signaling differs among the virus species. These findings highlight the complex and divergent effects of *Circoviridae* family capsid proteins on IFN-β signaling.

## 2. Materials and Methods

### 2.1. Plasmids

The IFN-Beta_pGL3 plasmid was a gift from Nicolas Manel (plasmid #102597; http://n2t.net/addgene:102597; accessed on 13 November 2024; RRID: Addgene_102597, Addgene, Watertown, MA, USA). pCAGGS–humanTRIF–Myc [19] and pCAGGS–pigTRIF–Myc [20], in which the pCAGGS vector (Addgene) encoded Myc-tagged human TRIF and pig TRIF, respectively, and the pRL–TK vector (Promega, Madison, WI, USA, Cat# E2241) were used to evaluate IFN-β signaling. The coding sequence of the *Circoviridae* capsid plasmids was synthesized according to the amino acid sequences deposited in GenBank with codon optimization to human cells (Integrated DNA Technologies, Inc., Coralville, IA, USA). The synthesized DNA sequence and details of the samples from which these sequences were derived are summarized in Table S1. Synthesized DNA was cloned into the pCAGGS–DsRed–monomer plasmid [21], which was prelinearized with *Age*I–HF (New England Biolabs (NEB), Ipswich, MA, USA, Cat# R3552L) and *Nhe*I–HF (NEB, Cat# R3131L) using an In-Fusion HD Cloning Kit (TaKaRa, Kusatsu, Japan, Cat# Z9633N). The plasmids were amplified using NEB 5–alpha F’ *Iq* competent *Escherichia coli* (NEB, Cat# C2992H) and extracted using the PureYield Plasmid Miniprep System (Promega, Cat# A1222). The sequences of all plasmids were verified using the SupreDye v3.1 Cycle Sequencing Kit (M&S TechnoSystems, Osaka, Japan, Cat# 063001) with the Spectrum Compact CE System (Promega).

To generate pCAGGS–DsRed–monomer plasmids expressing capsid proteins with deletions or chimeric capsid proteins between PCV2 and BFDV capsid proteins, each fragment was generated with PrimeSTAR^®^ GXL DNA Polymerase (TaKaRa, Cat# R050A). The primers used for PCR amplification and the amino acid sequences are listed in Table S2 and Table S3, respectively. The amplified fragments were cloned into the pCAGGS–DsRed–monomer plasmid, and the sequences of all plasmids were verified as previously described.

### 2.2. Cell Culture

Lenti-X 293T cells (TaKaRa, Cat# Z2180N) were cultured in Dulbecco’s modified Eagle’s medium (Nacalai Tesque, Kyoto, Japan, Cat# 08458-16) supplemented with 10% fetal bovine serum (Cytiva, Marlborough, MA, USA, Cat# SH30396) and 1× penicillin–streptomycin (Nacalai Tesque, Cat# 09367-34) at 37 °C in a humidified incubator with 5% CO_2_.

### 2.3. Nuclear Localization of Circoviridae Capsid Proteins

Lenti-X 293T cells were seeded into a 24-well plate (FUJIFILM Wako Pure Chemical, Osaka, Japan, Cat# 630-28441) at 1.25 × 10^5^ cells/well, cultured overnight, and transfected with 500 ng of pCAGGS–DsRed–monomer *Circoviridae* capsid plasmids using TransIT-293 Transfection Reagent (TaKaRa, Cat# V2700) in Opti-MEM (Thermo Fisher Scientific, Waltham, MA, USA, Cat# 31985062). NucBlue Live ReadyProbes Reagent (Hoechst 33342, Thermo Fisher Scientific, Cat# R37605) was used to probe the nucleus. The localization of *Circoviridae* capsid proteins was evaluated 48 h after transfection using the EVOS M7000 Imaging System (Thermo Fisher Scientific).

### 2.4. IFN-β Luciferase Reporter Assay

Lenti-X 293T cells were seeded in a 96-well plate (FUJIFILM Wako Pure Chemical, Cat# 63528511) at 3 × 10^4^ cells/well, cultured overnight, and co-transfected with 50 ng of pCAGGS–DsRed–monomer *Circoviridae* capsid plasmids, 2.5 ng of IFN-Beta_pGL3 plasmid, 45 ng of pRL–TK plasmid, and 2.5 ng of pCAGGS–humanTRIF–Myc or pCAGGS–pigTRIF–Myc plasmid using TransIT-293 Transfection Reagent. Two days after transfection, the luciferase activity was measured using a Dual-Glo Luciferase assay (Promega, Cat# E2920) and a GloMax Explorer Multimode Microplate Reader to investigate the effect of *Circoviridae* capsid proteins on IFN-β signaling. Firefly luciferase activities were normalized according to *Renilla* luciferase activities. The percentage relative activities were calculated by comparing the normalized luciferase data of *Circoviridae* capsid protein plasmid-transfected cells and empty plasmid-transfected cells. The assays were repeated at least three times. The data are presented as the mean ± SD of one representative experiment.

### 2.5. Western Blotting

The cellular pellets of Lenti-X 293T cells were lysed with 2× Bolt LDS sample buffer (Thermo Fisher Scientific, Cat# B0008) containing 2% β-mercaptoethanol (Bio-Rad, Hercules, CA, USA, Cat# 1610710) and incubated at 70 °C for 10 min. The expression of *Circoviridae* capsid proteins was assessed using SimpleWestern Abby (ProteinSimple, San Jose, CA, USA) with mouse monoclonal anti–DsRed–monomer antibody (clone OTI4c8, Thermo Fisher Scientific, Cat# TA180084) and Anti–Mouse Detection Module (ProteinSimple, Cat# DM-002). The amount of input protein was measured using the Total Protein Detection Module (ProteinSimple, Cat# DM-TP01). The predicted sizes of the DsRed–monomer *Circoviridae* capsid proteins were calculated according to the Protein Molecular Weight website (https://www.bioinformatics.org/sms/prot_mw.html, accessed on 13 November 2024).

### 2.6. Phylogenic Analysis of Circoviridae Capsid Proteins

A phylogenetic tree was constructed using 68 *Circoviridae* capsid protein sequences obtained from GenBank. The tree was created using the MUSCLE algorithm within MEGA11 software v11.0.13 [22]. We subsequently constructed a phylogenetic tree from the aligned amino acid sequences obtained from public databases. Evolutionary analysis was performed using the maximum likelihood and neighbor-joining methods and employing the Jones–Taylor–Thornton matrix-based model.

### 2.7. Calculation of Capsid Identity in the Circoviridae Family Among Animal Species

The level of conservation among the capsid proteins within the *Circoviridae* family was examined using The Sequence Manipulation Suite (http://imed.med.ucm.es/Tools/SMS/ident_sim.html, accessed on 13 November 2024), as described in Table S4.

### 2.8. Statistical Analysis

Unless stated otherwise, the data are presented as the mean ± SD of six measurements from a single assay, reflecting the results from at least three independent experiments. Variations in the relative values between the circovirus capsid proteins and the plasmid encoding only DsRed–monomer were analyzed using one-way analysis of variance (ANOVA), followed by Dunnett’s post hoc test for multiple comparisons. A statistically significant difference was indicated by *p* ≤ 0.05. All statistical analyses were performed using Prism 10 software v10.4.1 for Mac (GraphPad Software, Boston, MA, USA).

## 3. Results

### 3.1. Divergent Effects of Five Circoviridae Capsid Proteins on IFN-β Signaling

**Figure 1:**
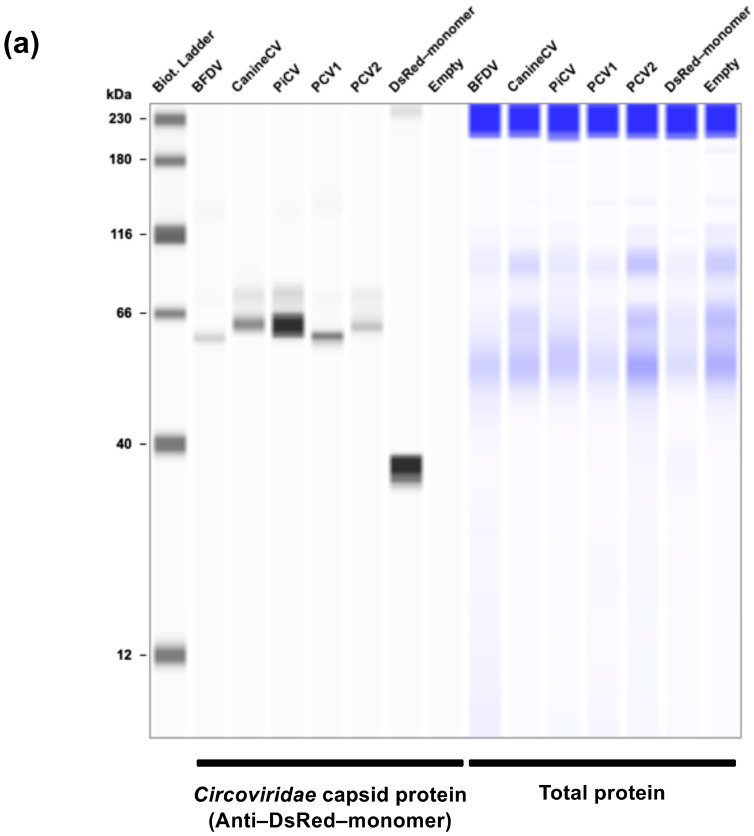

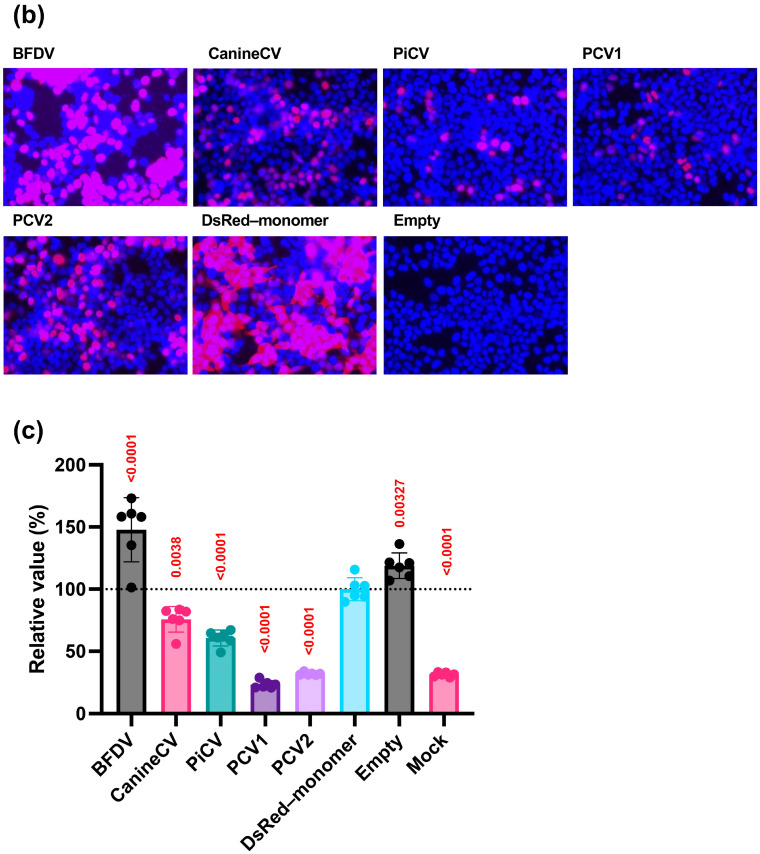
Divergent effects of five *Circoviridae* capsid proteins on human TRIF-mediated IFN-β signaling. (**a**) Expression of five *Circoviridae* capsid proteins in Lenti-X 293-T cells. The expected DsRed–monomer-tagged *Circoviridae* capsid proteins had expected sizes of 53.07–57.54 kDa according to the Protein Molecular Weight website (https://www.bioinformatics.org/sms/prot_mw.html, accessed on 13 November 2024). (**b**) Cellular localization of the five DsRed–monomer-tagged *Circoviridae* capsid proteins. The red signal indicates DsRed–monomer and the blue signal indicates Hoechst 33342. (**c**) Effects of the five *Circoviridae* capsid proteins on human TRIF-mediated IFN-β signaling as determined using the IFN-β luciferase reporter assay. Differences between cells transfected with plasmids expressing *Circoviridae* capsid proteins and DsRed–monomer plasmids were examined by one-way ANOVA followed by Dunnett’s multiple comparison test. *p*-values less than 0.05 are highlighted in red.

Immunosuppression is a common pathology observed in *Circoviridae*-related infections, including PCVAD and PBFD [12,23]. However, the molecular basis of immunosuppression remains unknown. Previous reports indicated that the PCV2 capsid protein localizes to the nucleus and inhibits IFN signaling [12]. To determine whether this feature is conserved throughout the *Circoviridae* family, we first performed experiments using capsid proteins derived from five *Circoviridae* viruses, specifically PCV1, PCV2, PiCV, CanineCV, and BFDV. We selected these five *Circoviridae* capsid proteins because PCV2, PiCV, CanineCV, and BFDV are clinically significant viruses in veterinary medicine, and PCV1 was included in the panel as a nonpathogenic virus. First, we tested the expression of DsRed–monomer-tagged *Circoviridae* capsid proteins in Lenti-X 293T cells by Western blotting. The expected molecular weights of DsRed–monomer-tagged *Circoviridae* capsid proteins ranged 53.1–57.5 kDa according to the Protein Molecular Weight website, and the results of Western blotting were consistent with the expected sizes (Figure 1a). Next, we tested the cellular localization of these capsid proteins. The PCV2 capsid protein localized to the nucleus, consistent with previous reports (Figure 1b) [17]. The other four capsid proteins also displayed nuclear localization (Figure 1b).

Next, we observed the effects of the five circoviruses on IFN-β signaling. Consistent with previous reports [15], PCV2 suppressed IFN-β signaling by up to 32.0% versus the control (DsRed–monomer, Figure 1c). Similarly, CanineCV, PiCV, and PCV1 inhibited IFN-β signaling by as much as 75.8%, 60.7%, and 23.5%, respectively. In sharp contrast, BFDV enhanced IFN-β signaling by up to 147% (Figure 1c).

### 3.2. Regions/Motifs Determining the Phenotypes of the BFDV and PCV2 Capsid Proteins

**Figure 2:**
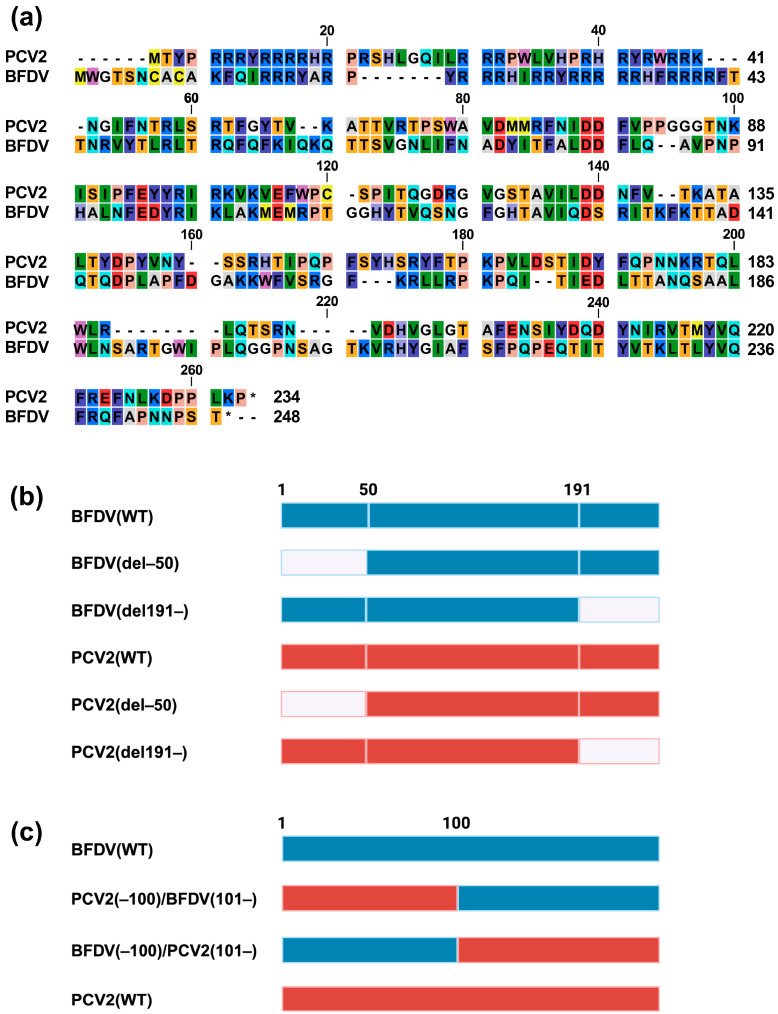

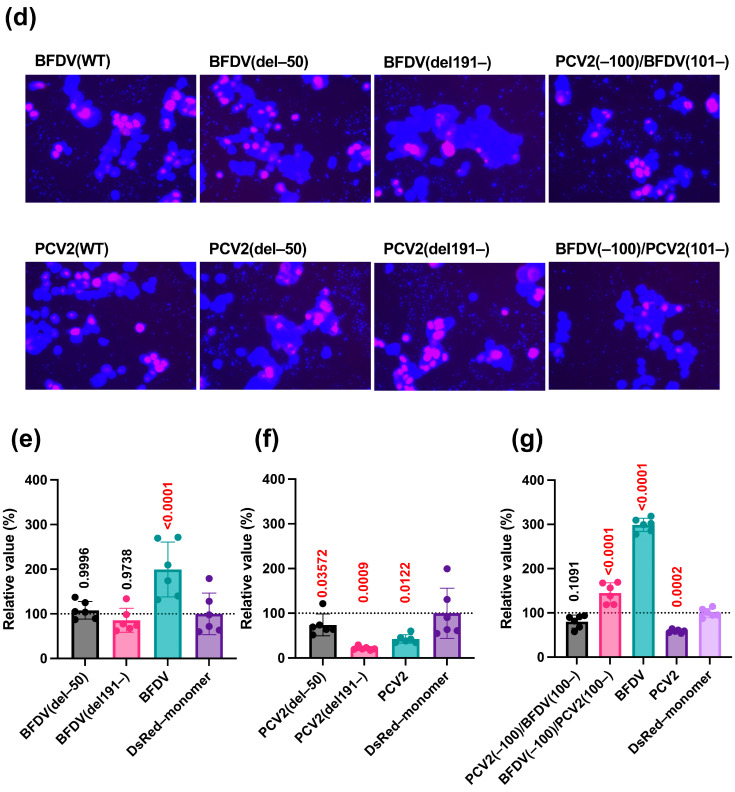
Phenotypes of deletion mutants of PCV2 and BFDV capsid proteins and chimeric proteins between PCV2 and BFDV capsid proteins. (**a**) Amino acid alignment of PCV2 and BFDV capsid proteins. (**b**) Schematic representation of the deletion mutants of PCV2 and BFDV capsid proteins. (**c**) Schematic representation of chimeric proteins between PCV2 and BFDV capsid proteins. The “*” represents the stop codon. (**d**) The cellular localization of the DsRed–monomer-tagged capsid proteins. The red signal indicates DsRed–monomer and the blue signal indicates Hoechst 33342. (**e**–**g**) Effects of capsid proteins on human TRIF-mediated IFN-β signaling. Relative value of the IFN-β luciferase reporter assay. Differences between cells transfected with plasmids expressing capsid proteins or DsRed–monomer plasmids were examined by one-way ANOVA followed by Dunnett’s multiple comparison test. *p*-values less than 0.05 are highlighted in red.

To determine the domains/motifs responsible for the opposing effects of BFDV and PCV2 on IFN-β signaling, we first created an alignment between BFDV and PCV2 (Figure 2a). Based on the alignment, we made mutant capsid proteins of BFDV and PCV2 (Figure 2b,c). First, we generated mutant capsid proteins with deletion of the N-terminal or C-terminal region (Figure 2b). Specifically, we generated plasmids expressing BFDV (del–50) and PCV2 (del–50) proteins with the deletion of 50 N-terminal amino acids because the N-terminus features a 41-amino-acid nuclear localization signal (NLS) responsible for nuclear localization [12].

Because the location of the NLS is unknown in the BFDV capsid protein, we used the cNLS Mapper website (https://nls-mapper.iab.keio.ac.jp/cgi-bin/NLS_Mapper_form.cgi, accessed on 13 November 2024) to identify the possible NLS. We found that 27 amino acid sequences starting from amino acid 208 in the C-terminus could be a possible NLS (Figure S1). Based on this simulation, we generated plasmids expressing BFDV (del191–) and PCV2 (del191–) proteins featuring the deletion of the C-terminus after amino acid 190. The nuclear localization of the PCV2 (del–50) protein was slightly weakened compared with that of the wild-type PCV2 (PCV2 (WT)) protein (Figure 2d). Although no effect on nuclear localization was observed with the BFDV (del–50) protein, we observed slightly weaker nuclear localization for the BFDV (del191–) protein compared with that of the wild-type BFDV (BFDV (WT)) protein.

Next, we examined the effect of the five mutant capsid proteins on IFN-β signaling. We observed that both BFDV (del–50) and BFDV (del191–) proteins lost the ability to enhance IFN-β signaling (Figure 2e). In the case of PCV2, although PCV2 (del–50) lost its suppressing effect, PCV2 (del191–) retained its suppressing effect (Figure 2f).

We further generated chimeric capsid proteins between BFDV and PCV2 (Figure 2c). Although both the PCV2 (–100)/BFDV (101–) and BFDV (–100)/PCV2 (101–) proteins localized to the nucleus (Figure 2d), the BFDV (–100)/PCV2 (101–) protein exhibited an enhancing effect similar to that of the BFDV (WT) protein (Figure 2g).

Taken together, although both BFDV and PCV2 capsid proteins localized to the nucleus, their localization was dependent on different regions/motifs. Furthermore, these viruses utilized different regions/motifs to modulate IFN-β signaling.

### 3.3. Conserved Nuclear Localization and Divergent Effects on IFN-β Signaling of Circoviridae Capsid Proteins

**Figure 3:**
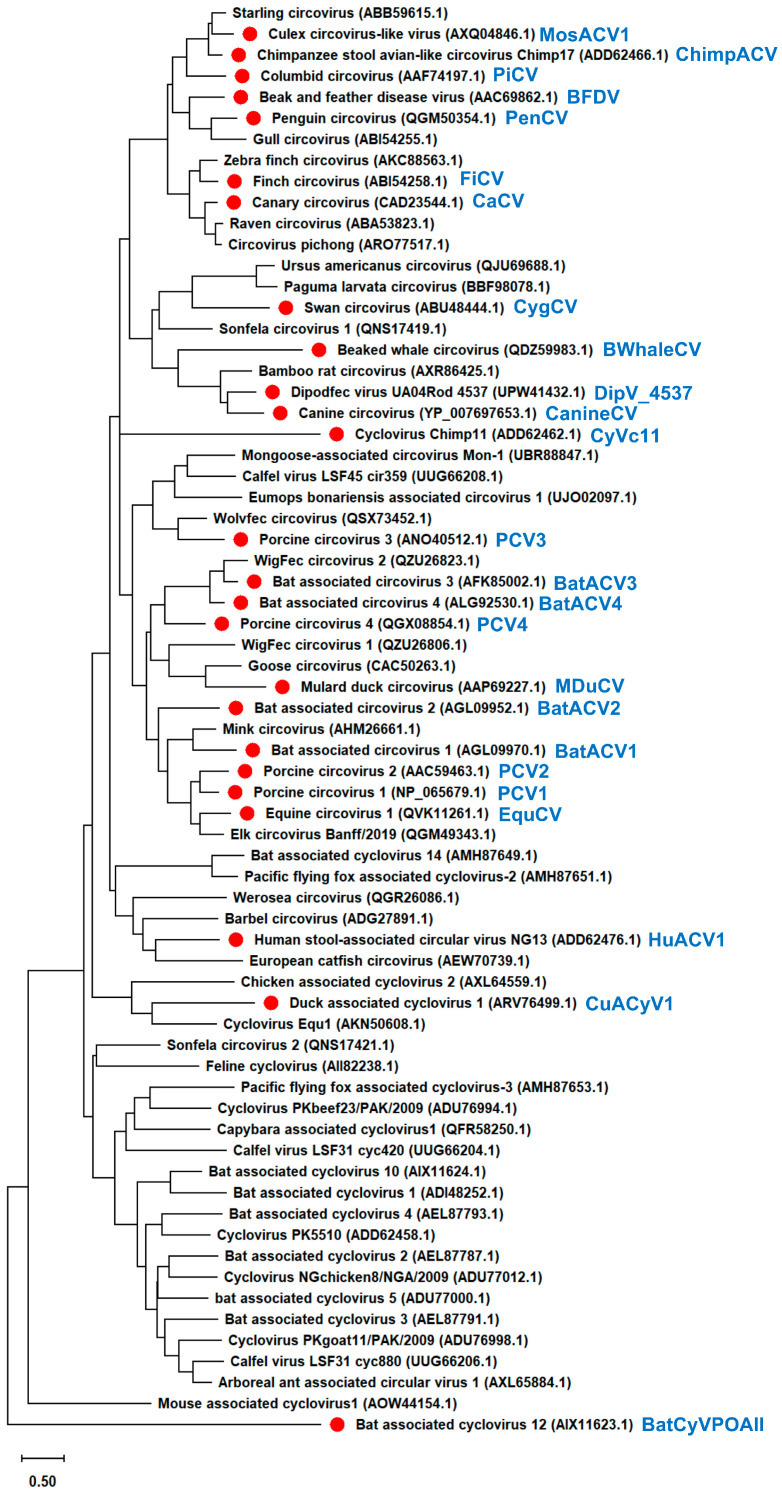
Phylogenetic tree of *Circoviridae* capsid proteins. The phylogenetic tree was constructed using MEGA software [22], and evolutionary analysis was conducted using the maximum likelihood and neighbor-joining methods based on the Jones–Taylor–Thornton matrix-based model with 1000 bootstrap replicates. In the phylogenetic tree, the capsid proteins characterized in this experiment are marked with red circles.

We demonstrated that the BFDV capsid protein had a distinct effect on IFN-β signaling (Figure 1c). To elucidate whether this phenotype is specific for the BFDV capsid protein, we expanded the analysis to include additional *Circoviridae* capsid proteins. We constructed a phylogenetic tree of 68 *Circoviridae* capsid proteins and generated plasmids encoding 25 *Circoviridae* capsid proteins representing each cluster (Figure 3). Although PCV1 and PCV2 capsid proteins were grouped into the same cluster, PCV3 and PCV4 were grouped into different clusters. Interestingly, all bat-associated circoviruses (BatACV) were categorized into different clusters (Figure 3).

**Figure 4:**
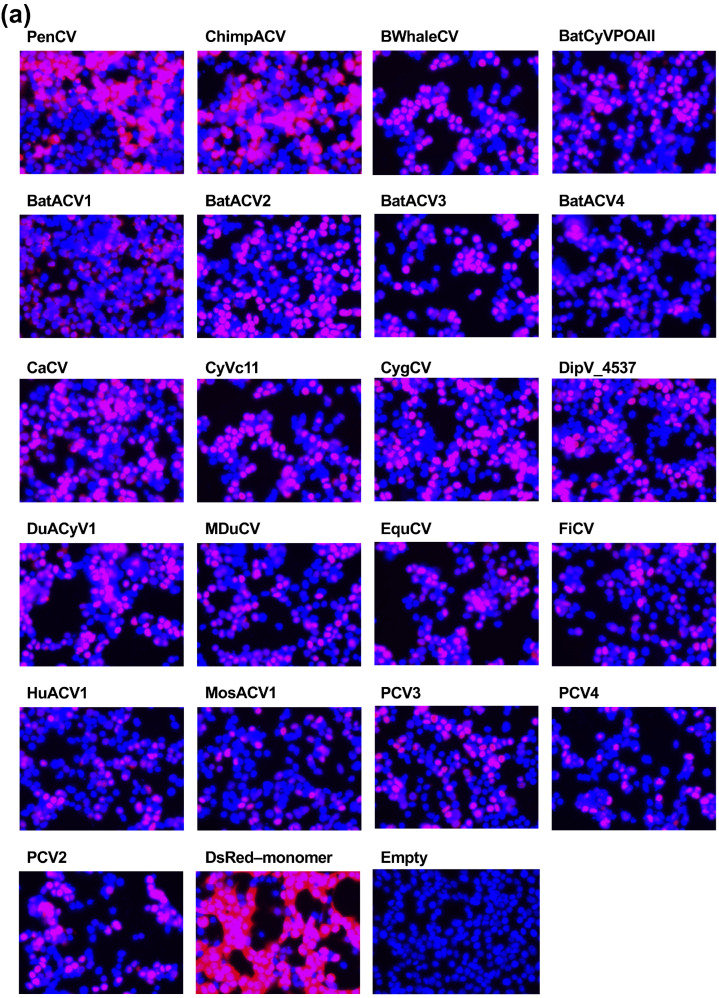

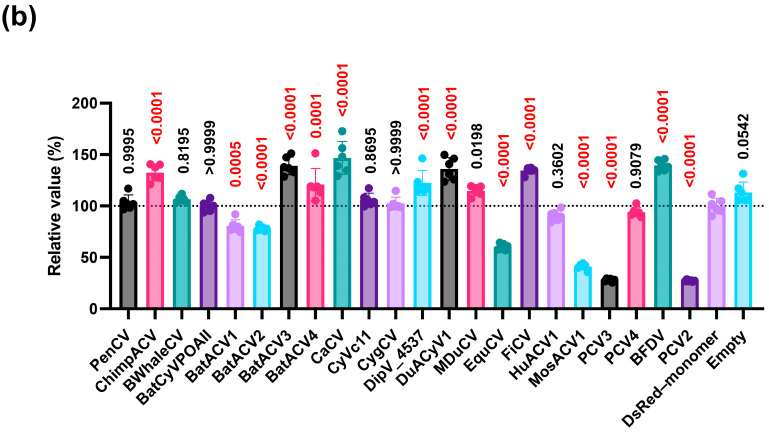
Effects of *Circoviridae* capsid proteins on human TRIF-mediated IFN-β signaling. (**a**) The cellular localization of DsRed–monomer-tagged *Circoviridae* capsid proteins. The red signal indicates DsRed–monomer and the blue signal indicates Hoechst 33342. (**b**) Effects of *Circoviridae* capsid proteins on human TRIF-mediated IFN-β signaling. The relative values determined by the IFN-β luciferase reporter assay are presented. Differences between cells transfected with plasmids expressing *Circoviridae* capsid proteins or DsRed–monomer plasmids were examined by one-way ANOVA followed by Dunnett’s multiple comparison test. *p*-values less than 0.05 are highlighted in red.

The nuclear localization of capsid proteins was conserved among the *Circoviridae* family (Figure 4a). In sharp contrast, we found a divergent effect on IFN-β signaling. Although capsid proteins from chimpanzee stool avian-like circovirus (ChimpACV), BatACV3, canary circovirus (CaCV), dipodfec virus UA04Rod_4537 (DipV_4537), mulard duck circovirus (MDuCV), and finch circovirus (FiCV) enhanced IFN-β signaling, IFN-β signaling was suppressed by BatACV1, BatACV2, equine circovirus 1 (EquCV), and Culex circovirus-like virus (MosACV1) capsid proteins (Figure 4b). These observations suggested that the enhancing effect on IFN-β signaling was not BFDV capsid protein-specific.

### 3.4. Effect of Circoviridae Capsid Proteins on IFN-β Signaling Induced by the Pig-Derived TRIF Protein

**Figure 5:**
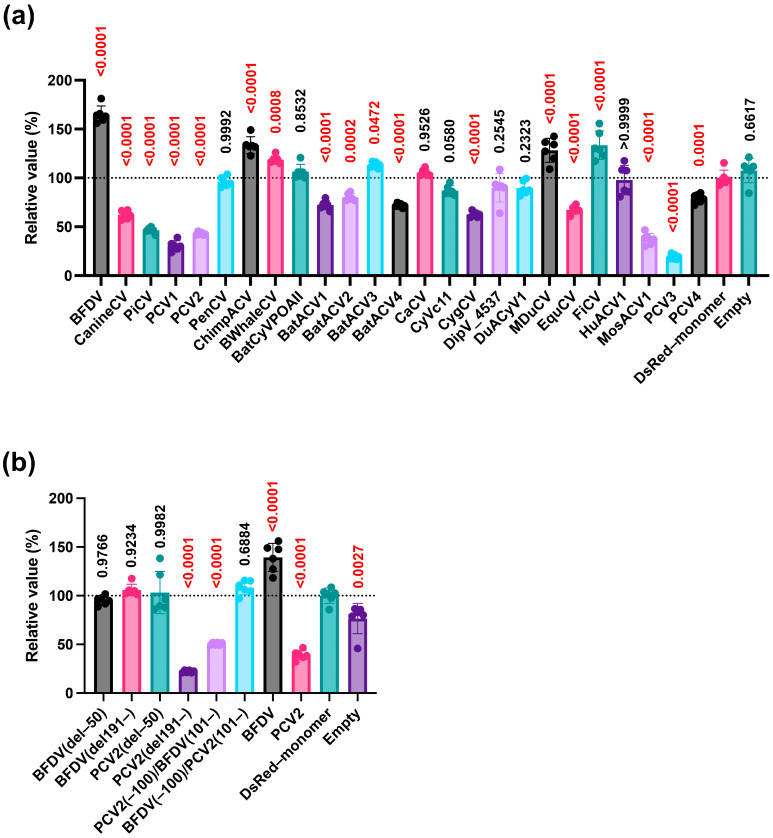
Effects of *Circoviridae* capsid proteins on pig TRIF-mediated IFN-β signaling. (**a**) Comparisons of the effects of capsid proteins from different *Circoviridae* species on pig TRIF-mediated IFN-β signaling. (**b**) Comparisons of the effects of deletion/chimeric constructs of *Circoviridae* capsid proteins on pig TRIF-mediated IFN-β signaling. The relative values determined by the IFN-β luciferase reporter assay are presented. Differences between cells transfected with plasmids expressing *Circoviridae* capsid proteins or DsRed–monomer plasmids were examined by one-way ANOVA followed by Dunnett’s multiple comparison test. *p*-values less than 0.05 are highlighted in red.

Because the aforementioned experiments were performed using human-derived TRIF protein (Figure 1c, Figure 2e,f,g and Figure 4b), we repeated the experiments using pig-derived TRIF protein. We evaluated the effects of 25 *Circoviridae* capsid proteins, including mutant capsid proteins, in Lenti-X 293-T cells. The results were similar to those obtained with human-derived TRIF protein (Figure 5a). However, although the PCV2 (–100)/BFDV (101–) protein did not affect IFN-β signaling induced by human-derived TRIF protein (Figure 2g), the PCV2 (–100)/BFDV (101–) protein suppressed IFN-β signaling induced by pig-derived TRIF protein (Figure 5b). This result suggests that the effects of *Circoviridae* capsid proteins differ depending on the source of TRIF.

## 4. Discussion

This study focused on immunosuppression, a prevalent pathology in *Circoviridae*-related infections, and we investigated the conservation of several functions of *Circoviridae* capsid proteins. Our findings revealed substantial divergence in the effects of different virus species on IFN signaling. This emphasizes the complex interactions within the *Circoviridae* family.

Our research began with a comprehensive examination of the intracellular localization of five *Circoviridae* capsid proteins derived from PCV1, PCV2, CanineCV, PiCV, and BFDV. We discovered that all capsid proteins consistently localized to the nucleus (Figure 1b). This thorough analysis was expanded using capsid proteins from genetically distant viruses within the *Circoviridae* family (Figure 3). The results revealed that all 25 *Circoviridae* capsid proteins localized to the nucleus (Figure 4a), further confirming the conserved nuclear localization of the capsid proteins.

The results using deletion mutants of the PCV2 capsid protein illustrated that nuclear localization was slightly weakened when the N-terminal 50 amino acids were deleted (Figure 2d). This result is consistent with a previous study revealing that the N-terminal 41 amino acids of the PCV2 capsid protein are associated with nuclear localization [17]. Conversely, the BFDV (del191–) protein exhibited weaker nuclear localization (Figure 2d), suggesting that the C-terminal region is responsible for the nuclear localization of the BFDV capsid protein. However, as the effect of the deletion in these regions was partial (Figure 2d), it is possible that other domains and/or motifs of the capsid proteins were involved in their nuclear localization. Thus, it will be intriguing to determine the impact of these regions on the localization of other *Circoviridae* capsid proteins. Further studies can address these points.

We examined the effects of *the Circoviridae* capsid proteins on IFN-β signaling, specifically examining five *Circoviridae* capsid proteins derived from PCV1, PCV2, CanineCV, PiCV, and BFDV (Figure 1c). Although PCV1 is considered nonpathogenic, PCV2, CanineCV, PiCV, and PCV1 capsid proteins suppressed IFN-β signaling (Figure 1c, Figures S2 and S3). Moreover, although the phenotype of the PCV2 capsid protein was consistent with previous findings [16], the BFDV capsid protein enhanced IFN-β signaling. These results suggest that immunosuppression, a common pathogenesis in *Circoviridae*-related infection, may be caused by a mechanism more complex than anticipated.

Our analysis using deletion mutants of capsid proteins suggested that the N- and C-termini of the BFDV capsid protein are required for its enhancing effect (Figure 2e). In the case of the PCV2 capsid protein, the C-terminus appeared dispensable for its inhibitory effects (Figure 2f). Further analysis using a chimeric protein between PCV2 and BFDV capsid proteins demonstrated that the BFDV (–100)/PCV2 (101–) protein had an enhancing effect, albeit weaker than that of the BFDV (WT) protein (Figure 2g). This suggests that the effect of the BFDV capsid protein was dominant over that of the PCV2 capsid protein. Furthermore, these results suggest that although the PCV2 capsid protein does not require the C-terminal region to suppress IFN-β signaling, the N- and C-termini are involved in the IFN-β-signaling-stimulating effect of the BFDV capsid protein, and the C-terminal region of the PCV2 capsid protein can complement the activity of its C-terminal region.

Our experiment using 25 *Circoviridae* capsid proteins revealed that the effects of these proteins on IFN-β signaling varied greatly among the virus species (Figure 1c, Figure 4b and Figure 5a). Similar to the PCV2 capsid protein, several *Circoviridae* capsid proteins such as PiCV, CanineCV, PCV1, PCV3, BatACV1, BatACV2, EquCV, and MosACV1 capsid proteins suppressed human TRIF-mediated IFN-β signaling (Figure 1c, Figure 4b and Figure S2). Contrarily, capsid proteins from ChimpACV, BatACV3, BatACV4, CaCV, DipV_4537, DuACyV1, and FiCV, similarly to the BFDV capsid protein, enhanced human TRIF-mediated IFN-β signaling (Figure 1c, Figure 4b and Figure S2). CaCV and FiCV, which have similar hosts and pathogenicity characteristics to BFDV, exerted a similar enhancing effect on human TRIF-mediated IFN-β signaling (Figure S2). Notably, 11 of the 25 viruses, namely BFDV, CanineCV, CaCV, PiCV, PCV2, PCV3, PCV4, EquCV, CygCV, CaCV, and FiCV, were associated with pathogenicity (Table S1). However, the associations of most of the other viruses with pathogenicity were not clarified (Table S1). Therefore, although we aimed to clarify the relationships of capsid proteins with pathogenicity, we could not identify a clear association, and further research is needed to address this point. It is possible that PCV2, PCV3, and PCV4 exhibited similar suppressing effects against pig TRIF-mediated IFN-β signaling because they were isolated from sows with PCVADs (Figure 5a and Figure S3, Table S5). However, among PCV1, PCV2, PCV3, and PCV4, only the PCV4 capsid protein failed to suppress human TRIF-mediated IFN-β signaling (Figure 4b). Considering that the PCV3 capsid protein was more distinct from the PCV2 capsid protein than the PCV4 capsid protein in the phylogenetic tree (Figure 3), this result supports our hypothesis that the effects of *Circoviridae* capsid proteins on IFN-β signaling are independent of genetic similarity. When we focused on *Circoviridae* capsid proteins that enhanced human TRIF-mediated IFN-β signaling (Figure 4b and Figure S2), ChimpACV, CaCV, BFDV, and FiCV capsid proteins were found to be genetically similar (Figure 3). However, PiCV, which is genetically similar to BFDV, suppressed human TRIF-mediated IFN-β signaling (Figure 1c and Figure S2), suggesting that the effect of capsid proteins on IFN-β signaling is not associated with the genetic distance (Figure 3, Figures S2 and S3).

This study had several limitations. First, we were unable to identify the regions responsible for the divergent effects of *Circoviridae* capsid proteins on IFN-β signaling. Second, we did not elucidate the correlation between the suppressing effect of capsid proteins on IFN-β signaling and clinical severity (Figures S2 and S3). Moreover, as PCV2 and BFDV infections induce immunosuppression, other viral proteins of BFDV might be responsible for its immunosuppressive effect. These factors should be investigated in future studies. Third, we used a luciferase-based reporter system in human-derived Lenti-X 293-T cells to test the effect on IFN-β signaling. As different results were obtained with several *Circoviridae* capsid proteins between human- and pig-derived TRIF proteins (Figure 4b and Figure 5a), it is worth testing TRIF proteins from other animal species. Furthermore, we must use cell lines derived from other animals, including pigs and parrots, to clarify the significance of PCV2 and BFDV capsid proteins on IFN-β signaling.

In summary, our study revealed the diverse effects of *Circoviridae* capsid proteins on IFN-β signaling, revealing that these effects were virus-species-specific. This emphasizes the intricate pathophysiology induced by viruses in the *Circoviridae* family. These findings also deepen our understanding of these viruses and provide a foundation for future research to develop effective treatments for animals infected by *Circoviridae* viruses.

## Data Availability

The original contributions presented in the study are included in the article/Supplementary Materials. Further inquiries can be directed to the corresponding author.

## Supplementary Materials

The following supporting information can be downloaded at https://www.mdpi.com/article/10.3390/pathogens14010068/s1: Figure S1: Possible NLS sequences in PCV2 and PBDFV capsid proteins; Figure S2: Classification of the impact on human TRIF-mediated IFN-β signaling (Suppressing, Enhancing, or No effect) based on the association with pathogenicity (Yes (red), No (blue), or Unknown (green); Figure S3: Classification of the impact on pig TRIF-mediated IFN-β signaling (Suppressing, Enhancing, or No effect) based on the association with pathogenicity (Yes (red), No (blue), or Unknown (green)); Table S1: Synthesized DNAs for generating plasmids encoding capsid proteins and the details of the samples from which these sequences were derived; Table S2: Primers used for generating plasmids encoding mutant *Circoviridae* capsid proteins; Table S3: Amino acid sequence of mutant *Circoviridae* capsid proteins; Table S4: Genetic distance of *Circoviridae* capsid based on the amino acid sequence; Table S5: Multiple comparisons of the effect on pig TRIF-mediated IFN-β signaling between PCV1, PCV2, PCV3, and PCV4 capsid proteins.
