# Supplementary material for: Divergent Effects of Circoviridae Capsid Proteins on Type I Interferon Signaling
Source: Pathogens. 2025 Jan 13;14(1):68. doi: 10.3390/pathogens14010068 (PMC11768430; doi:10.3390/pathogens14010068)
Supplement: Supplementary file 1 [file pathogens-14-00068-s001.zip › 241215 Supplementary Table 2nd_revise_AS8 Final.pdf]

Supplementary Table S1. **Synthesized DNAs for generating plasmids encoding capsid protein and the details of the samples from which these sequences were derived**

**Capsid protein**	**Sample origin**	**Association with pathogenicity**	**Codon-optimized DNA sequence**
Porcine circovirus 2 (PCV2)<br>Accession #<br>AAC59463.1	Lung, lymph node, spleen and tonsil tissue from pigs affected by PMWS<br><br>(Hamel et al., 1998)	**Yes**	AGGATCTCAGACCGGT<u>ATG</u>ACGTATCCAAGGAGGCGTTACCG<br>CAGAAGAAGACACCGCCCCCGCAGCCATCTTGGCCAGATCCT<br>CCGCCGCCGCCCCTGGCTCGTCCACCCCCGCCACCGCTACCG<br>TTGGAGAAGGAAAAATGGCATCTTCAACACCCGCCTCTCCCG<br>CACCTTCGGATATACTGTCAAGGCTACCACAGTCAGAACGCC<br>CTCCTGGGCGGTGGACATGATGAGATTTAATATTGACGACTTT<br>GTTCCCCCGGGAGGGGGGACCAACAAAATCTCTATACCCTTT<br>GAATACTACAGAATAAGAAAGGTTAAGGTTGAATTCTGGCCC<br>TGCTCCCCCATCACCCAGGGTGATAGGGGAGTGGGCTCCACT<br>GCTGTTATTCTAGATGATAACTTTGTAACAAAGGCCACAGCCC<br>TAACCTATGACCCATATGTAAACTACTCCTCCCGCCATACAATC<br>CCCCAACCCTTCTCCTACCACTCCCGTTACTTCACACCCAAAC<br>CTGTTCTTGACTCCACTATTGATTACTTCCAACCAAATAACAA<br>AAGGACTCAGCTTTGGCTGAGGCTACAAACCTCTAGAAATGT<br>GGACCACGTAGGCCTCGGCACTGCGTTCGAAAACAGTATATA<br>CGACCAGGACTACAATATCCGTGTAACCATGTATGTACAATTC<br>AGAGAATTTAATCTTAAAGACCCCCCACTTAAACCC<u>TAA</u>GCT<br>AGCAGATCTTTTT

Porcine circovirus 1 (PCV1)<br>Accession #<br>NP065679.1	Porcine (*Sus scrofa*) kidney-derived PK-15 cell line<br><br>(Niagro et al., 1998)	**No**	AGGATCTCAGACCGGTATGACCTGGCCGCGACGGCGGTACA<br>GACGCAGGAGAACCAGACCACGCTCCCATCTCGGAAATATA<br>CTCCGCCGCCGCCCGTATCTCGCCCATCCAGCCTTCCGGAAC<br>AGGTACCGCTGGCGACGAAAAACCGGGATCTTCAATTCCCG<br>CTTGTCAACGGAGCTCGTCCTTACGATCAAGGGGGGGTATA<br>GTCAACCAAGTTGGAATGTCAATTATCTGAAATTTAATATCG<br>GCCAGTTTCTCCCGCCTTCAGGGGGCACGAATCCGCTGCCCC<br>TCCCCTTTCAGTACTACCGCATCCGAAAGGCAAAGTATGAGT<br>TTTATCCCCGCGATCCCATAACTTCTAATCAAAGGGGCGTCG<br>GTTCAACGGTAGTCATCCTGGATGCAAACTTCGTTACTCCGT<br>CCACAAATTTGGCTTATGACCCTTATATCAACTACTCTTCAC<br>GGCATACTATAAGACAGCCTTTCACCTATCATTCAAGATATT<br>TTACGCCTAAACCTGAACTCGACCAGACCATAGACTGGTTCC<br>ATCCAAATAACAAAAGGAACCAACTTTGGTTGCACTTGAAC<br>ACACATACTAATGTTGAGCATACGGGTCTCGGGTATGCTCTC<br>CAGAACGCCGCAACCGCGCAGAACTACGTTGTGCGCCTCAC<br>GATATATGTCCAGTTCAGGGAATTCATTCTGAAAGATCTTTG<br>AGCTAGCAGATCTTTTT
Psittacine beak and feather disease virus (BFDV)<br>Accession #<br>AAC69862.1	Feathers from PBFD-affected cockatoos<br><br>(Bassami et al., 1998)	**Yes**	AGGATCTCAGACCGGTATGTGGGGAACCTCTAATTGTGCATG<br>CGCTAAATTCCAGATTCGGAGGCGCTATGCTCGACCATACAG<br>ACGGAGACATATAAGACGCTACAGGCGGAGGCGCAGGCATTT<br>TCGACGGAGGAGATTCACAACTAATCGAGTCTATACGTTGAG<br>ACTTACCCGGCAGTTTCAGTTCAAGATACAGAAACAAACTAC<br>CAGCGTTGGTAACCTTATTTTTAACGCCGATTATATAACATTTG<br>CCCTGGACGACTTCCTCCAGGCAGTACCTAACCCACATGCGT<br>TGAACTTCGAGGATTATCGCATCAAACTCGCGAAGATGGAAA<br>TGCGCCCAACGGGGGGACATTATACGGTCCAATCTAATGGATT<br>TGGACATACAGCAGTAATTCAAGACTCTAGAATAACCAAGTT<br>CAAAACAACTGCGGATCAAACACAAGATCCGCTCGCCCCGTT<br>TGACGGCGCTAAAAAGTGGTTCGTATCACGCGGATTCAAGAG<br>GCTGTTGCGACCTAAACCTCAGATAACCATTGAGGATTTGAC<br>TACGGCTAACCAATCAGCTGCTCTGTGGCTTAATTCAGCCCGC<br>ACTGGCTGGATTCCACTGCAAGGTGGCCCCAACTCAGCTGGC<br>ACCAAAGTACGGCATTATGGTATCGCTTTTTCTTTTCCGCAAC<br>CGGAACAGACCATCACATACGTGACCAAACTGACACTTTACG<br>TCCAGTTCAGACAATTCGCCCCCAATAACCCGTCCACGTAAG<br>CTAGCAGATCTTTTT

Columbid circovirus<br>(Pigeon circovirus:<br>PiCV)<br>Accession #<br>AAF74197.1	Bursa of Fabricius of pigeon showing histopathological lesions typical for circoviral infections<br><br>(Mankertz et al., 2000)	**Yes**	AGGATCTCAGACCGGTATGCGGCGAAGAAGGTTTAGACGGC<br>GGCGAGCACCAATTCGACGCCGCAGGATTAGGAGGAGGCGC<br>ACACGCCTCTCACGAAACATTCGCGGGCACCGCCGGTCTAG<br>TCGCATTTACTATTTTCGGCTTCGCAGAAAAGATAAAATAAC<br>CTTGACGCAAGCGACAAACGATTTCAAATTCGGCACGGGCA<br>TATTCACATTCAAACTCGCGGACGTATTGACGGTAGGTCTTA<br>ATGCACCAACGCTGAAGGTGCCCTTTGAAGATTACCAGATA<br>GCTCTCGTTAAAGTAGAGATGAGGCCGTTGGGTGTTGACATC<br>ACTACCTGGAAGGGATTCGGCCATACCGTTCCGATGTACGAT<br>GCTCGCCTCAAAACGTTTCAAGGACAGGTCGATTTGGGTGAT<br>GATCCCCTGATGGACTTCGATGGGGCGCGCAAATGGGATTT<br>GAGGAAGGGGTTCAAGCGGCTTATCAGGCCGCGACCCCAGC<br>TGACCATCGCCGATTTGGCAACCGCGAACCAAAGCGCCGCC<br>ACGTGGTTTTCCGGGAGAAACCAGTGGATACCATTGCAGGT<br>TTCAGGTAACTCACTGTTTCCTCAGAAAGTTAATCATTACGG<br>GTTGGCATTCTCCTACCTCCAGCCCCAACCAGACCCCATGTA<br>CTACGAGTGCGAGGTGACGTTCTATGTCAAATTCCGGCAATT<br>CGCCTGGACAACGCTTAATGTTCCTCCGACACCTAATATTGA<br>AGGCATGGAGCTTATGCACATCTGCAACGGGGATTGCAACC<br>AATGCTTCGCCGATGCGCTTGATCCGGACTCTGCTGTAGATA<br>GTGAATGAGCTAGCAGATCTTTTT

Canine circovirus<br>(CanineCV)<br>Accession #<br>YP007697653.1	Tissues from dogs (*Canis lupus familiaris*) with vasculitis and hemorrhage<br><br>(Li et al., 2013)	**Yes**	AGGATCTCAGACCGGTATGAGGGTCAGACGACATGCGCGAG<br>CGTCACGGAGATCATACAGGACGCGGCCGTTGAATAGGTAC<br>CGACGACGCAGGCAGAATCGGTTTAAGCTGTTCCATTTGCG<br>GTTGCGCCGAACCCTTACAGCAGACTGGCCGACTGCACCCG<br>TGAAACCGACTAACGATCCACAGACAGAGACTCCACTGCTC<br>TGGAACTTCGATCATCTTTCATTCAAACTGACAGATTTTTTG<br>CAAGCTAGTCATGGGACTGGAGACTTTCAACACCTCCCGCCC<br>TTCCGGTTCTATAAATTTAAAAAAGTGTATATACGAGCTCGC<br>TGGATAAACTGGCCACGAACACTTATGGAGAATGTCCTTGG<br>TCGGACGGCATTGGATTTGGACGGAGAAGATCAGGGTAGAG<br>GCAACGCGACGCGCTCCCATTTGGATCCTGGTACTGTGCCGG<br>GCAGACTGGAACCACCGAAAGACCCCAACAAGGCGCCGTTC<br>ATCTATGATCCCTTGCAAGACCGCTCCTCCTCCAGATCCTTC<br>AATATGGCGAGCGGGTTTAAAAGGGGGCTGACTCCGAAGCC<br>GATGTTTACTCAAGACATCGCCAGTCCGTCAGCGACAGCAC<br>CATGGCTTACAAGGGGGACTCCATGGGTCAGCGTCATCCAA<br>GGTGCTAACATGGTATGGAACGGGTTGTCTATTTCACTGAGA<br>CAAATGAAGGACATGAGACCTACAACGCCAGACACCTCAAC<br>ATCTCAGATTCCTCAGGTTCAGTATGACATCAGCGCATACAT<br>CGCCTTCAAAGAATTTGATTATGAAACAGGTCGACAACTGT<br>AAGCTAGCAGATCTTTTT
Porcine circovirus 3<br>(PCV3)<br>Accession #<br>ANO40512.1	Skin, kidney, lung, and lymph node localized in typical PDNS lesions, including necrotizing vasculitis, glomerulonephritis, granulomatous lymphadenitis, and bronchointerstitial pneumonia from sows that died with PDNS-like clinical signs, and aborted fetuses of these sows<br><br>(Palinski et al., 2017)	**Yes**	AGGATCTCAGACCGGTATGCGCCACAGGGCCATATTTCGAA<br>GACGACCTCGGCCTCGCCGCAGACGCCGACATAGAAGACGG<br>TACGTTAGGCGGAAGCTGTTCATTAGAAGGCCTACCGCTGG<br>GACGTACTATACGAAAAAGTACTCCACCATGAACGTCATCT<br>CAGTCGGCACGCCGCAGAACAATAAACCGTGGCACGCAAAT<br>CATTTTATCACCAGGCTCAATGAGTGGGAGACCGCAATAAG<br>CTTTGAATACTACAAGATACTCAAGATGAAGGTTACCCTGA<br>GCCCTGTCATCTCTCCCGCACAACAGACCAAAACCATGTTCG<br>GGCATACCGCAATCGACCTCGATGGCGCTTGGACCACTAAC<br>ACATGGTTGCAAGACGACCCGTATGCGGAATCATCTACCAG<br>AAAAGTCATGACGAGCAAGAAAAAGCATTCAAGGTATTTTA<br>CTCCCAAACCGATCCTCGCTGGAACCACAAGTGCCCATCCA<br>GGTCAGTCCCTTTTTTTCTTCAGTAAACCAGCACCCTGGCTC<br>AACGCCTATGACCCCACAGCCCAATGGGGTGCACTGTTGTG<br>GTCAATATATGGGACCGAAAAGACCGGGATGACTGATTTTT<br>ATGGATACCTGGAGGTGTGTATCCAATACACTAGCGATCTCT<br>AAGCTAGCAGATCTTTTT

Porcine circovirus 4 (PCV4)<br>Accession #<br>QGX08854.1	Nasal swabs and serum consisting of lung, spleen and kidney from the pigs had severe clinical signs including respiratory disease and diarrhea and a small percentage of them had skin lesions suggestive of porcine dermatitis and nephropathy syndrome (PDNS).<br><br>(Zhang et al., 2020)	**Yes**	AGGATCTCAGACCGGTATGCCAATACGCTCCCGATATTCAA<br>GACGCCGCCGCAACAGGCGAAACCAACGAAGGCGAGGGCT<br>GTGGCCTCGGGCAAATCGGCGGAGGTACCGCTGGCGCCGAA<br>AAAACGGTATTTTTCATGCGCGGTTCATGCGAGAAGTGACA<br>CTCAGTGTAAGTTCATTTTCTACTCCTAGCTGGAACGTGGGC<br>CATTATGATTTCAAACTCAAGGATTTTATCCCAAAGGGTCCT<br>GGGACTATAGTTAACCTTTACTCCCTGCCGTTTGCGTACTAC<br>AGAATAAGAAAGATTAAAGTAGAATTTTTGCCTTTGAACGG<br>GATTAATTCCAACCGAACCTATTCATCTACGGCGATCCAGCT<br>CGACGGCGACTATGTGGGCGAAGGTAAGAACCAAACCTATG<br>ATGTACTGGCCAATCATTCCAGTCGGCATGGATTTACAAACA<br>TTGCTAGACACTCCCGGTACTTCACGCCTAAACCACAGGACC<br>CATCCGGTGAAACGCACACGCTTCACTTTCAGCCTAATAATA<br>AACGGAATCAATGGTGGATCTCTATGGCCGACCAAGACTTG<br>GTGCATCACGGCTTGCAGTACAGCATTCAAAACAGCAACTT<br>CGTACAGGTGTGGACTGTACGGTTCACGATGTATGTCCAATT<br>CAGGGAATTCGACCTTGTCAATTATCCTAAGCAAGGATAAG<br>CTAGCAGATCTTTTT
Bat associated circovirus 1<br>(BatACV1)<br>Accession #<br>AGL09970.1	Organs of the thorax and abdomen of bats<br><br>(He et al., 2013)	**Unknown**	AGGATCTCAGACCGGTATGCCTATTAGAAGACGGTCACGCT<br>ATAGCAGGCGGCGGCGCTGGAGGAGGAATACCAGGCGACG<br>CCGGGTAGCCCGAGGGGCTTACAGATGGCGCAGGAAGAATG<br>GAATAATCAACGTCAGGCTTAGTGCCACCAAAGATTGGACT<br>ATGGCATCTACAACTGCGGAGGGTTACAATGTCGCGAGGCT<br>CGAAGTGAATCTGAGACAATTCATGCCCGCAGGTCCGGGTA<br>GTGCTATAAACACCAAGTCAATCCCATGGGCCTATTATCGGA<br>TCAGGAAGATGAAGTTCGAGATCCTGCCTAAAATGATTCCT<br>GCTCAGACGCCATATCGATACGGGTCTACAGCTATATACCTG<br>GGCATGCAGGCTCCTGCTCCCACCCAAGGTAAAACGTACGA<br>TCCTCATTTGAAGCATGTTAAACAGAATATGAGCGGTCTGAT<br>AACGGATCAATTGAAAAGGTATTTTACGCCCAAGCCCGACC<br>TGGATAGCATAACTTCTACTGCTTGGTTTCAGCCCAATAACA<br>AGGCAAATCAGGTTTGGATTAACATGACCAATGACAACATA<br>ACTCATGGGCAAGTTGGATGGTCTATGGAACGGATCTCTAA<br>CATGGCCCAAAACTTTAAGATAAGAGTGACGTTGTATGTAC<br>AATTCCGAGAGTTCAACCTTATAGACTACCCCGCACAAGCTC<br>CACTGCTGGTAGACGAAGAGCCAAGTGAATAAGCTAGCAGA<br>TCTTTTT

Bat associated circovirus 2 (BatACV2)<br>Accession #<br>AGL09952.1	Organs of the thorax and abdomen of bats<br><br>(He et al., 2013)	**Unknown**	AGGATCTCAGACCGGTATGGTTTACCGCCGGAGGCGCGGTC<br>GAGGTAGGCGAGCGCGGCCCATGAGTAGTCTGGGGAGACTT<br>CTCTACAGAAAGCCGTGGCTTATGCATCCGCGGTTCCGAGCA<br>AGATATCGCTGGCGACGGAAGAATGGGATCACAAACCTGCG<br>CCTTACCCGGCAGGTGGAACTCTGGGTCCCCAAGGACGCGG<br>CGAACGCTTCTTTCTATGTCAACCACTACACATTTGATCTTG<br>ATGATTTCATCCCAGCAGGCACGCAACTCAACTCCAGTCCTC<br>TGCCTTTTAAGTATTATAGAATTAGAAAAGTAAAAGTTGAGT<br>TTCAACCCCGCTTGCCCATTACCTCCCCCTTTCGGGGGTATG<br>GAAGCACAGTGCCAATCCTCGATGGGGCGTTTGTAACGCCC<br>GCAACTGGCGAGAGTGATCCCATATGGGACCCATATATAAA<br>TTTTTCTGGGAGGCACGTGATCAGAACCCCAGCCTGGTATCA<br>TAAGAGATATTTTACACCTAAACCACTTATTGACGGGAATAC<br>TGGTTTCTTTCAACCTAATAATAAGCAGAATGCGTTGTGGTT<br>TCCTAATAAGCAGGGCCAGAATATACAGTGGTCTGGACTGG<br>GATTTGCCATGCAGAAGGGGAACGAAGCGTACAACTACCAA<br>GTACGATTCACCTTGTACGTACAGTTCCGGGAGTTTGACCTT<br>TTCAATAACAAGTACACAGCACACATGGATGTCCCTCTCTAA<br>GCTAGCAGATCTTTTT
Bat associated circovirus 3 (BatACV3)<br>Accession #<br>AFK85002.1	Pharyngeal swab and anal swab from 11 insectivorous bat species from six provinces in China for metagenomic analyses<br><br>(Wu et al., 2012)	**Unknown**	AGGATCTCAGACCGGTATGCCAAGGTCTACGAGACATAGGT<br>GGAGGCGGAACCAATGGTTCAAAAGGTGGCGCCAGAGGCG<br>AAGAAGGGGGCATACAAGGGGACGGAGAAGGTACAGGAAC<br>AAAGTCGGCATATACAATTTCAGGTTCCGCGCCATCACTACA<br>ATGACTATCAATAAAAATTCCAACCAAGGTTACTTTACTTAT<br>ACACTCAACGGTTCTGTGCCTACAGCCTTTGCTAATTATTTT<br>GACATGTATAGGATTGCGAAAGTACGCGTTCAATGGCTTCCT<br>ATGGTGTCTATTAGCGAGGTGCGCGCTTGGGGTGCAACCGTT<br>ATTGACCTCACGGGGCGCGACACAACAGTGCCCTCAACAGG<br>GCGGACTGACTTTACAATAGATGATTCCACTCGCCGGTTGTG<br>GAACCCTACAAGAATACACTCCAGGTACTTTACGCCCAAGC<br>CCGAAATTCAGATCAGATCTAACTCAGAGGCGGTGCAGCCA<br>AATAACCCGAGAAACCAGCTTTGGATCGATAGCCGAGACAA<br>GGACGTTAAGCATCACGGTATAGCTTATTACTTCCACCCCGA<br>CGACATCGGGGATGATGTCTATAAGTTCTCATATATCGTGAC<br>GTATTATTTCCAGTTTAGGCAGTTCGCGGGTAGCCAGGCGCC<br>TTCCGTTTAAGCTAGCAGATCTTTTT

Bat associated circovirus 4<br>(BatACV4)<br>Accession #<br>ALG92530.1	Respiratory fluid from Brazilian free-tailed bats (*Tadarida brasiliensis*)<br><br>(F. E. S. Lima et al., 2015)	**Unknown**	AGGATCTCAGACCGGTATGCCACGATCCCGCCGCCACAGAT<br>GGCGGAGGAACCAATGGTTTAAACGCTGGAGAAGATCCAGG<br>CGCAGGGGGCATACTTTCGGTACTCGCCGCTGGAGACAAAA<br>AAATGGCATCTACAATTTCAAATTCAAGGCTAATTCTCTCCA<br>GACCGTAAACAAAACCAGCAATCAAGGGTACTTCACATTCT<br>CCCTTGCTGCTGCATGTCCGCGAGCTTTTTCAACATATTTGA<br>CATGTACCGAATCCGAAAGGTCCGAGTTCAGTGGCTTCCCAT<br>GTCAGGTATCAACGAGCCTCGCGTTTGGGGTGCAACAATTAT<br>AGACCTCACCGGCCGGGACACAACCCTGCCCTCCACGGGTA<br>ATACGGACTTTACGATAGACGATGTAACTCGACGAATCTGG<br>AACCCAACGCGGCTCCATTCACGCTACTTCACTCCTAAACCG<br>GAAATAGCGATCAAGACTGTATCATCAGAGGCAATTCAGCC<br>CAACAATCCTCGGAACCAACTGTGGCTCGATGCGAAATACC<br>CCGATGCAAAGCATCACGGGTGCGCCTTTTTTTTCGGGCAAA<br>CTGATCTTGGTGATGAATCTTATAAGTTCCAGTTCCTTGTAA<br>CCTACTACATCCAATTCCGCCAATTCGCGGGCTCACTCGCAC<br>CGAGCTAAGCTAGCAGATCTTTTT
Equine circovirus 1<br>(EquCV)<br>Accession #<br>QVK11261.1	Blood of a febrile horse with hepatitis	**Yes**	AGGATCTCAGACCGGTATGACGTGGCCTCGCAGGCGACGGA<br>GATATCGGCGAAGAAGAAGTCATCTCGGGTACATCCTTCGA<br>GCCCGCCCCCATCTGGTACATCCAAGATTCCGACGAGCGTAC<br>AGATGGCGACGGAAAAACGGAATATTTCAAGCGCGACTCAG<br>CCGCAATCTCAACTACACGATAGCTTCCCAGAGCAGCTTGCC<br>TTCTTGGAATATCTCCCAAATTAGGTTTAATATCGGGGCACA<br>CCTGGTGCCAACAGGATCAACCTACAACCCGTTGCCCTTGCC<br>ATTTCAATACTATCGGATAAGGAAGGCGAAAGTTGAATTGT<br>TCCCCCAAAGTCCAATCACTTCTGGCGAGCGCGGTGTGGGAT<br>CCACCGCCATTGTGCTGGACGGAGCGTACCGGCAGAATCTT<br>ACTGCTGCGACGTTCGACCCGTACCTCAATTATAGCACCAGA<br>CATGTTATACGACAGCCATTCACATATCACTCTCGATACTTT<br>ACCCCTAAGCCTCAGATAGAACTTGAGGGTTGGTTCCAACC<br>GAATTCCAAAAGAAATCAAATGTGGCTCAATCTTGACACTC<br>AGAATAATCTCGATCATCATGGACTTGGGTTGGCACTCGCTA<br>ATAGCTCATCTGCTCAGTCCTATACAATTCGGATAACACTTT<br>ATGTACAATTCAGGGAATTTATCTTGCAAGATCCTAATGAAG<br>TATAAGCTAGCAGATCTTTTT

Mulard duck circovirus (MDuCV)<br>Accession #<br>AAP69227.1	Homogenates of bursal tissue from mulard ducks showing a feathering disorder, poor body condition and low weight<br><br>(Hattermann et al., 2003)	**Yes**	AGGATCTCAGACCGGTATGCGAGGAAGGTCATACCGACGCG<br>CCTACAGAGGGCGGCGGAAAAGGAGAGGTCTTCGACGCCGA<br>TTCAGACGCCGGCGACTGAGAATTGCACGGCCACGAAGAAG<br>ATTTAGTGTAGTAACATACAAGGTTACAAGAAACACAGTGT<br>TCGGCTTCTTCGGCAGTCAGACGGGCCCAACCGCAGCGGGG<br>AAATGGCAAAGCTTGTCTCTCGAGGACGGTGCCCAATACAC<br>TGACCCACCAGCACGGGGTAATAACATTTGCGGGCTGAATA<br>TGCGCTGGGCGATGTTCGGTGACACAAATAGCTACATGACT<br>GGCAGTACACCCAATTATCATTATCCATACGATTATTATATG<br>ATTAAAGGCGTTGCAATTACCTTGCGACCCGCGTACAATATC<br>TATCAAAAGAGCAAAACACAAGGCAGCACAGTAATTGACAA<br>AGACGGACAGATCGTCAAAACGGCTACTACTGGTTGGTCCA<br>TTGATCCCTACGGAAGCACTTCATCACGCCGGACATGGGAC<br>CCTAGTCGAGTTCACAGGCGGTACTTTGTACCAAAACCAATC<br>ATTCAGGGTGCTGGAGAGGGTACAAAATACTCTACATTTTTC<br>CTCGGGGGTCGAAATTTTACATGGATAAACTGTACCCAAGA<br>CCAGGTCGTTCATTACGGAATGGGAATGAGCCTGCGGAAGC<br>CCGACAATACGACGGGGGTCAATGCCCAGTATGATATCGAA<br>GCCCAATTTACTTTCTACATAAAATTTGGACAGTTTACCGGC<br>TTCTAAGCTAGCAGATCTTTTT
Swan circovirus (CygCV)<br>Accession #<br>ABU48444.1	Mixture of liver and spleen from mute swans (*Cygnus olor*) found dead during an epidemic of sudden death in Germany in 2006<br><br>(Halami et al., 2008)	**Yes**	AGGATCTCAGACCGGTATGTCACTCTCCCGCGCGGGAAGTA<br>GGAGACTCTACCATAGAAGACGCCGCGCCTATTACCGAAGA<br>AGGCGCTACGGGCGCCGACGCCTTAGGATACCGAGAATCAG<br>GCGCAGATTTACCGTGTTTCATGTTAAACAAACTCAGAACAT<br>TCCGTTTACTTTTTGGAGTTCTGCAACCCCCGACCCTACCGA<br>CAAATGGATGTCATTGAGTCTGGAATCCAATTCAGCCACACT<br>CAACAAGCCCAAACCAGGCCTTAACCTGCGGTTTGCTATGTT<br>TGGTGATAGACTTCCAGGAAGTGGCAATGCGTACCACTACC<br>CTTTTGACTATTACATGATAAAACTGGTAAAAGTTGAGCTCC<br>GGCCTGCGTTCAACCCGTTCCAAAAGCAGAAAACTCAAGGG<br>AGCACTTACATCGACAAAGAAGGGGATATAACCGAAGTACC<br>TGCGACGACACCTTGGAGTGTCGATCCATATGCTGCAATGTC<br>ATCCCGCAAAACGTGGACACCAGACCGCTACCACAAACGAG<br>TATTCATCCCGAAGCCCACGATACAGAGCTCTCAGACGACA<br>GGAACGCGGTTTTCAACCTGGTTCGTGCCGGGAAGACGGAA<br>TATGTGGATAAATAGTACCCAGGACCAGGTAATACACTATG<br>GTATGGGTATGTCACTTCGAGCCCCTGCCGAAGATGCTGGTG

			CATACCCTGTCGAGTGTACCGTCACCTTCTATATTGCATTCG<br>GACAATGGACGGGATTGAGCCCTTAAGCTAGCAGATCTTTTT
Duck associated cyclovirus 1 (DuACyV1) Accession # ARV76499.1	Cloacal swab of *Anas platyrhynchos*	**Unknown**	AGGATCTCAGACCGGTATGGCTAATTATAGAAGAAAAAAGA<br>AGTACGGCAGAAGATATCGGCCACGGCCATTGAGAGTACGC<br>CTGTATAATCCAAAACGGAATAGGCTCCGACGAACGCAAAG<br>GCGGCTCAAAAATGACAAATACTTCACGAGGTTTATGTCAT<br>GGACATCAGTAGATGTTGATCCTAAAATCGGGCAGGGATGG<br>TACTTGGCCCCCAACTATAAGTCCAACCCGGGGTTTCTGCAA<br>CTGGCCGAGCAATACTCAGAGTTTAAAATACACAAGGTAGC<br>GGCAAGGATCACTCCGGGCCTGAACGAAACCTATCTCGCAG<br>AGCAGGGGCTGCGATATGCTTACTGTCCGTGGCAACAAGAC<br>AACGGTGGCAGTCCGTCCACAAATGATATAGTGAGTAACGG<br>AACTGACCTGACGTATGGAAAACTTATGAATGTCCCGTGGG<br>CAAAATCCAAGCGGCTCCATCAATCTGCTACCATAGTCTACA<br>AACCTAAGTGCAATCAGGCGATAGCTTTTGCGCCCAGGACA<br>TCCAGTGGAGCAAACGGTAATCAAAGGGTCATTTCATTCCCT<br>TGGGTAACGACCCAAGCCATGTCCATGGGGGCTCTCCCTGGT<br>ATGTCTGGAATTATGCTCGCATTTGACCAACAAGACCCACTT<br>AGCGTTATTGGAAAGCCATTGAGGGTGCAAGTCGAGTTCTTT<br>CTTTATGTGACGTTCCGCAAGCGAAGAAACCCAATGCTTCAG<br>ACGCCCACACCCTTTGTCATGGACCAGTAAGCTAGCAGATCT<br>TTTT
Beaked whale circovirus (BWhaleCV) Accession # QDZ59983.1	Samples from *Indopacetus pacificus* (The details of the samples are unclear)	**Unknown**	AGGATCTCAGACCGGTATGGTCAGACGGTATAGACGGCACC<br>GACCTTATTTTCGCCGGAGGAGACGCTACCACGGTTATAGG<br>AAACGCTTCAATCGGAGGCGACGCTTTCGCAAACCACGGCT<br>GTTTCACTTTCGATTTGAACGAACATGGTGCGCGCAATACCG<br>GAACCAAGATCTCCATCAGCCGTTTTGCGCCGCAGGATGGT<br>GGACTGATCGCTTCAGCCTGACACTGCGGCAGCTTCTTCAAT<br>CCAACCGGGCGGTTGGTCAGACTGATTGGGTCCCCCCCTTCC<br>AGGAGTTTTGCATTAAAAAAGTAGTTGTCCAATGGACCCCC<br>GTAGCTGAGCCAAATTATTCTCTCGAAAACCTGCCTGGTCAT<br>ACTGCGACAGACCTTCTTGGCGAAGATAGGCAGAAGAAATT<br>CGGACCCACCCCAGATGAGACTCAAAAACCGCCTATTTATC<br>CGGGGCTCAAGACCGAACCGCCTCTTGAAAATCGATCTAGT<br>ATGAAACATGCCAACTTTAGATTTCCAGTGAAGCGAATTTTC<br>AGACCCGTTCCGGCACTTCCTGCCGACTGCAAGCCACAGGA<br>GGATCCCTCTGTATGCGGAAGGTGGTTTTCAAGAAATAATA<br>AATGGCTCACGATCCGATTGAACATGGACACGGAGTGGGAT

			GGTATTTCATGGGATTTTAAACAGCCTTGTTTGGGGCCTACG<br>GTGCTGAATATAAGAGTGACAGCGTATGTCGCGTTTCGGGA<br>ATTTAATTATTGGCGCGGTTTCTCCATCTGTACTCAAACGGA<br>TTGTAAACCCTGTTATCATCCGCAAGGGCAGTGCGACGACCT<br>TACTTGCAATGTACAGAAACTCAATCTTGGTCGAGGGGAGA<br>GTAGATAAGCTAGCAGATCTTTTT
Dipodfec virus<br>UA04Rod_4537<br>(DipV_4537)<br>Accession #<br>UPW41432.1	Feces of *Dipodomys merriami*	**Unknown**	AGGATCTCAGACCGGTATGACTAGGCTCACGTCCCTGACAA<br>CGTCTTCAATCTTTAGGTTGCGGAGGCGACGGCCTAGACGGC<br>GACTGGGGAACAGGCCCTACGGGCTCCGATCTCGGAGCTTT<br>CGCCGCCGAAGGAGGTTTAGGAGAAATCGCAGACGCCATCC<br>ACGCCGAACATTCCACCTTCGCTTCCGCCAGGTAGTAACCAG<br>CGACTGGCCAACAGCTCCAACGACTACGCCTTCCGACGGCA<br>CGTCCGGCAAGGAAACACCACTTCAATGGAACTTCGACCAC<br>ATGAGCTTTTCCCTCAATCACTTTCTTCAAATTGGACACGGT<br>ACCAGTAGCTACCAGCATCAACCCCCATTCAGGTATTACAG<br>ATTTAAGAAAATCGTGGTTAAGGGGACATGGATCAATTGGA<br>CGTCCCAGTATTTCGAGAATGTAATTGGCCATACGGCTCTCG<br>ACCTCGACGGTGAAGACAAGAACCGGGGAAACGTCAATCGG<br>TCAAATCTCGACCCAGCGGAACATGCAGCTAGGTTGCGATC<br>CGACCCTACTAAGGCACCTTTCAACTATGATCCTCTCCAAGA<br>TCGCACGTCTGCAAGGAAGTTCAACATACGCAGGGGATTCA<br>CTCGAGTGTTGCGACCTAAACTGGAGATAACGCAACAGGTC<br>CAGACTCCTAAAATGGAGTTCCATTGGCTTACTAAGGGGAC<br>CCCGTGGGTAAGCGTGCGAGAAGGATCAAACATGCCGTGGA<br>ACGGACTGTCTATCAGTCTTCGCCAGATGAAAAACCCCACA<br>ACTGAGACACCCGATCCGCCAATCCCGCAAATACAGTATGA<br>TATCAGCGCCTACGTCGAGTTTAAGGAATTCGACTACGAAA<br>CATCAAAACAACTTTAAGCTAGCAGATCTTTTT
Penguin circovirus<br>(PenCV)<br>Accession #<br>QGM50354.1	Nest of *Pygoscelis adeliae*	**Unknown**	AGGATCTCAGACCGGTATGAGCTCTAACAGGAGGGGTCACC<br>GGACAAACAGGATCTATCTTTTCCGCTTCAAGCGACAATTCG<br>AGTTTAATCTTTCAAAAGAGACTACGCCTGGGCAATTGGAGT<br>TTGATAGTGATTACCTGACCTTCACACTTAATGATTTTATCG<br>GTGTGGAAGGAGTACCCCAACAATGGCCTTTTGAGGATTAT<br>CGGATACCACTCGCTAAAGTAGAACTGAAGTTGCGCGGAGA<br>CATGATGACCATCGGCCACGGTATAGGTCACACGGTTCCAA<br>TACAGGACTCCAGAATCCGGGATTTTAAGCTCAAGAATCAG<br>AAGCAAGACCCACTGGCAAATTGGGATGGAGCACGCCAGTG<br>GCTTCCGAACCGCGGTTTTAAGCGGATAGTGCGGCCAAAAC

			CTCAAATAACTGTTCAGGACTTGACGGCTACGAATCTTACCG<br>GATCTCTTTTTCTCAATTCAAGTCGCTCAGGGTGGCTTCCCTT<br>GCAGCTCCCCAATGGTACCACTAGAGATGGCGCTAAGATCG<br>TACATTATGGTCTTGCGTGGAGCTGGCCTCAGCCAGTCAAGG<br>ATCTGACCTACATGGCGGAGGTTACCATTTACGTGACCTTTC<br>GGCAGATGGCGTCTACACTGAAGAACATGTTGCTGTTGAAT<br>AACAATGCGGAATGTTTGAATATCGCAAACGATGAGGACGA<br>GCTCAATGAATATGCGGAATCAATGGAGAACACAGTATAAG<br>CTAGCAGATCTTTTT
Canary circovirus (CaCV)<br>Accession #<br>CAD23544.1	Organ homogenates from adult canaries that had died after a short illness characterized by dullness, anorexia, lethargy and feather disorder<br><br>(Todd et al., 2001)	**Yes**	AGGATCTCAGACCGGTATGTGGCTGACATTCAACCAGGTTG<br>CCCGGCGCCGCAGGCCCCTCGCCCCGCGCAGAAGACGCTGG<br>AGGAGACGGTATTGGCGCAGAAGGCGGATTCCGGCTAACAG<br>GAGAGGCCACCGAACTAACCGAGTATATAGATTCAGGTTCG<br>TTCGAGAATTTGGCCAGGTACTGCAAAAGGGAACGGGGGA<br>TCACAGCTCAGTTTTGGGACCGATGGGATAAACATTATTCTC<br>GACGACTTCCTTGACTGGGGAACTATTAATTGGAGGCTCCCG<br>TTCGAGGATTATCGAATTAGGTTGGCTAAGGTGGAAATGCG<br>CCCCCTGAATGAATCCTGGGAAGAGTGGAAAGGGTTCGGTC<br>ACAACGTACCCATTCAGGACAATCACCTGGAGGATTTCTTCA<br>AAAAGACACGGTTGGACGCGGACCCACTTGCCAATTGGGAC<br>GGGGCACGAAAGTGGGATCTGAGGAAAGGTTTTAAGAGACT<br>TTTTAAACCAAGACCTCAGCTTTCTGTCACCGACACGGACGC<br>GGCGAATGTAACTGCTGCGCTCTGGCTGAATAATCCTAAGTC<br>TCTCTGGATACCCATCATGAAAAAATCCGACCAAAACCTGC<br>CCTCCTCAGGCACGCGGGTCAAGCATTATGGTCTGGCTTTTT<br>CATGGCCAGAGCCCACACCAAATCAAATGGACTATCAGGTA<br>AAGGTTACGATCTACTGTGAGTTTCGGCAAATGAATCTTACC<br>CATTTGGCCACCCCGAAGTAAGCTAGCAGATCTTTTT
Finch circovirus (FiCV)<br>Accession #<br>ABI54258.1	Bursa of Fabricius of 10-week-old Gouldian finch (*Chloebia gouldiae*) infected with circovirus, which has nasal discharge, dyspnoea, anorexia and depression, and a herring gull (*Larus argentatus*) infected with circovirus<br><br>(Todd et al., 2007)	**Yes**	AGGATCTCAGACCGGTATGCTGACTGCCACCTGGAACCATTT<br>CCTCATACAACGCAGAATGCGCCATTGGCGACGACGCCGCC<br>ATCTTAGGATGCGCCGCAGACATTGGCGGAGGCGACGCAGG<br>ATCCCCGCGAACAGACGCGGGCATAGGACCAATAGGATCTA<br>CCGCATAAAATTGGTACGCCAGTATCAACAAATCCTCGGAA<br>AGGCAGAAAACGCGGGAGCCTTCCGATTTGGAACGGATGGC<br>GTAACAATCATACTCAAAGATTTCTTTCCAGAAGGTAAGGA<br>ACTCCCCTTCGAGGATTACAGGATCCGATTGGCAAAAGTAG<br>AAGGTATCCCCCGGAGAGAGGATTGGACTCACTGGGAAGGG

			GCTGGTGTAACTGTACCTATTCAGGATCAACACCTCCGCGAT<br>TTTTTTAAAAAGACACGACTTACCGTTGACCCCCTTGCAAAC<br>TGGGATGGCGCTCGCAAATGGACACTGAGAAAAGGGTTCAA<br>AAGGCTTTTTAGGCCTCGCCCGCAACTTAGTGTCACCGATAC<br>CGACACAGCAAATACGACCGCCGCCTTGTGGTTGAACAATC<br>CTCGGGGGGCTTGGATTCCCCTTATGAAAAAAGGGGAAACA<br>ACGGCAAGCACGACTAGTGGTACCCGCGTCAATCATTACGG<br>GCTGGCCTTTTCATTCCCGGAGCCTTGGCCAGCAGATTTTGA<br>GATGCAAATAAAGGCCACGATCTACGTTGAGTTTAGGCAGC<br>TCAATATGACACACATGAACTAAGCTAGCAGATCTTTTT
Chimpanzee stool avian-like circovirus Chimp17 (ChimpACV) Accession # ADD62466.1	Stool of chimpanzee<br><br>(Li et al., 2010a)	**Unknown**	AGGATCTCAGACCGGTATGAATCGAATATATCTTTTCCGCCT<br>CAGACACAGTGTAAAGGTTACTCTGCAGAGAGACGAAAGCG<br>GAGCCTTCAAATATGGAACGGATGCATTCACATTTACCCTTG<br>AAGATGTACTTAAAAGAGATGGCGGGCAGCTCAAGTTGCCC<br>TTCGAGGACTACACTATTGTCTTGGTCAAGGTGGAAATGCGC<br>CCATTGGGAGAATCCGTTACTATCTGGAAGGGGTTTGGACA<br>CACTGTACCCATACAAGATGCTAGACTGACGAACTTCAAAA<br>AGAAAGCGGGTTTGCAGGACGATCCGCTTGCTAATTTTGAC<br>GGAGCCAAAAAATGGGACCAGAGATGGGGTTTCAAGCGACT<br>GCTCCGGCCTCGCCCGCAGTTGGTTAGCACTGATCTGAGCAC<br>TGCGAATGAAGTAGCGCCAATCTGGCTCAACTCCCAGAGGA<br>GCTTCTGGATTCCTTTGCAACAGACAGCTCAAAGAATAGCGC<br>CAGAGAAGATAAATCACTATGGACTCGCGTTTTCCTACCTGC<br>AGCCCTCTCCTGAAGATGAGTTTTATCAGGCAGAAATCACAT<br>TTTATATGAAATTCCGCCAATTTGCCTGGACTACCCTTAATG<br>ATCCTCCGACCCCTAATTGGCAGGAGCAGCTCCCGCCTAACA<br>TGGAGCTCATGAATATCATAGATGAAGATGAGGGAGTTGAT<br>TTCCAGTAAGCTAGCAGATCTTTTT
Culex circovirus-like virus (MosACV1) Accession # AXQ04846.1	Mosquitos homogenate<br><br>(Sadeghi et al., 2018)	**Unknown**	AGGATCTCAGACCGGTATGCGGCCAATAGGGGAGCAATCTA<br>CTCTTTGGAAAGGGTTCGGACATACGGTGCCTATAACAGAC<br>CCCCGCCTGAATATGTTTGGGAAAAAGACAGGTTTGCTGGA<br>AGATCCCTTGCAAAACTTTGATGGTGCGAAGAAATGGGAAC<br>AAAGACGAGGCTTCAAGCGCCTCCTTCGCCCGAAACCCCAA<br>CTGATGATTTCAGATCTGGCAACAGCAAATCAATTGGCTTAC<br>ACCTGGTTTTCCAATCAGCGAAATCAGTGGATACCACTCCAG<br>ACGACTGGTCAACAGATCGCACCTACAAAAGTAAATTTCTA<br>TGGACTCGCTTACTCATACCTGCAACCCCAACCTGATGATAT<br>GCACTACGAAGCTGAAATCACTTTCTATGTGAAATTCCGCCA

			ATTCGCATGGACGGGCCTCGACAATCCGCCTAATCCCCACAT<br>AGAAAACAACCTGCCCAACCTGGACCTGTTGCACGTTTGCG<br>ACGGGGACTGTAACACTTGCTTCGCTACCTCTTTGGATCCTG<br>AGTCTATCGTTGAGAGCGATGCTGAG<u>TAA</u>GCTAGCAGATCTT<br>TTT
Human stool-associated circular virus NG13 (HuACV1)<br>Accession #<br>ADD62476.1	Stool of human<br><br>(Li et al., 2010a)	**Unknown**	AGGATCTCAGACCGGT<u>ATG</u>GGCGGCAGGAGGAGACAACGA<br>CGACCAGATCCGAGGCGGGGGCGCCGACGCGCTAGAACGTT<br>TCTGCCAGCCCGGCTCTACGGACAGACGTACAGTACCAGGC<br>TGATTAAGCAGGATACTTTTCGAGTGGACGCTGCGGGTAAA<br>GGCGTCGGATTGACGTTTACCCTTGGGGAGTTTCTTACTGGA<br>CAAATTACCTGGGACTACTACAGAATTAACACGATTGTCGTA<br>ACCTTCTTGCCACAAATCAACCCAACTATGCCCATCGACCAG<br>GGTACCGGGGGTGGCGGCTTCAATGAGACAAGTGTTGATTT<br>CGATGACGCAACACCTCCTACGTCTAAAATTTCCATGGAGA<br>ACTGGAACAATACCAAATTCTGGAGGAATGACAGGAAGTTT<br>ACAATCAAGTTTCGACCAGTGTTTCATAGACTTGTTGCAACT<br>TCCGCAACATCTACGACGCCGGTTTCAGCAGAATTTAACCAA<br>CGCCGGAATAGAGGCGTATGGTTGAACTCAGCTTATAAGGA<br>TATTCCTCATTTCGGCTTGAAGACTTTCTTTGTTAATAATTTT<br>ACAAATCCGGCCCAGAACACGATCATTTATCAAATACTGAT<br>AAAAGCATACGTGTCATTCAAGAGGCCAATTTGGGTAGGGA<br>CCACCACCGAAAACCAA<u>TAA</u>GCTAGCAGATCTTTTT
Bat associated cyclovirus 12 (BatCyVPOAII)<br>Accession #<br>AIX11623.1	Stool of *Molossus molossus* and *Tadarida brasiliensis*<br><br>(F. E. de S. Lima et al., 2015)	**Unknown**	AGGATCTCAGACCGGT<u>ATG</u>TCAAACCCGACAGTACGGCGGT<br>TTGTATTTACGTGGAACAACTACACCTCCGAGGATTATGACA<br>AATGCTGCGAGTTCATCAAATCAAATTGTAGATACGGTATA<br>GTTGGCAAAGAGATTGCGCCCACAACTGGATGTCCACACCT<br>TCAGGGGTTCTGCAACTTGCATAAACCTATGAGGTTCGGGGC<br>CATCAAAAAACACCTGCACAACAGCATCCACATCGAGAAAG<br>CTAATGGCAGTGATGAAGATAATAAGAAATATTGCTCTAAG<br>AGTGGGGAGACTTTCGAGCAGGGACATCCACACAAACAGGG<br>GGAACGGACGGACCTGCAATCCTGTATTAGTGATATACAAT<br>CCGGCGCGACGATCAAAAAAATTGCTGAGGACCATCCGGCG<br>GTGTTCATTCGCTACCATCGGGGGATTCGAGAGTACATACGA<br>ACGGTCATGCCCATCAAGGAAAGGGATTTTAAGACGGAGGT<br>ATATTACTATTGGGGCCCGCCCGGTAGCGGCAAGAGCAAGC<br>GGGCTCTTGAAGAGGCGAAACAAAGAGGTACATCAATTTAC<br>TATAAGCCACGCGGACTCTGGTGGGACGGATACCAACAACA<br>TGACTGCGTGATAATCGACGACTTTTATGGCTGGATAAAGTA

			CGACGAGATGTTGAAAATAATGGATAGGTACCCCTATAAAG<br>TCCAGATTAAGGGCGGTTTCGAAGAATTTACTTCTAAGTATA<br>TATGGATTACGTCTAACGTGGATACGGACGACCTCTACAAAT<br>TCATAGGGTATAAAACTGATGCGCTCGAACGCCGAATCACG<br>AATAAAGAATACATGGATTAAGCTAGCAGATCTTTTT
Cyclovirus Chimp11<br>(CyVc11)<br>Accession #<br>ADD62462.1	Stool of chimpanzee<br><br>(Li et al., 2010b)	**Unknown**	AGGATCTCAGACCGGTATGGCATTCAAAAGATACTTTAGACG<br>AAGAAGAAGGGTACGGAAACCAGTGCGCCGCTTCAGAAGGC<br>GCTACCGAATGAGAAGGCGCGTCCTCCGATCAAAACCAGGTA<br>ATATGTTGACAAAACTGACAAAGATTACGACACTGTCAGTGG<br>AGAACAATATAAATGCAACCTGGAGCTGCAGTTTTAAGATGG<br>GGGACTTCACAGAATATGGTAGGCTGGCTCCAAATTTCGAGA<br>CTGTAAAGCTGAATAAAGTAGTCGTGAGAGTACAGCCGTTGC<br>AAAACGTTGCCAATAATTCTACATCTAGCGTCCCCGCCTACGT<br>AGTCGTGCCGTGGCATTACAACATAGCACTTCCAAAAGACTT<br>CGCTTCTTATCTTCGGATAGATAAACACAAGCTCCGAGCTCAG<br>ACCGTAGGGACTTCAATGAGTTTTGTACCAAACATCGTTACA<br>GTAGGCGTGGCGAACGAGGGTGCAAATCCCACTGGAAGAAA<br>CATTACTTGGAAACCGACTTTGGAATGCCTGGGCGTGGACAT<br>TAATATACCAAGGGTGTACTGCGGAGCCATCTGTTTCCAAGG<br>GCAGCCTGACATGGAGGGAAGGAAGACTGCTTTCAATATAAT<br>AACTGACGTATACTGTACATTCCGAAATCAGAACACTATGAA<br>GGTCTAAGCTAGCAGATCTTTTT

Supplementary Table S2. Primers used for generating plasmids encoding mutant *Circoviridae* capsid proteins

Capsid-F	AGGATCTCAGACCGGTATG
Capsid-R	AAAAAGATCTGCTAGCTTA
PCV2(101–)-F	CAAACTCGTTAAGGTTGAATTCTGGCC
BFDV(–101)-R	ACCTTAACGAGTTTGATGCGATAATCCT
BFDV(101–)-F	AAGAAAGGCGAAGATGGAAATGCGCCC
PCV2(–101)-R	ATCTTCGCCTTTCTTATTCTGTAGTATT
PCV2(del–50)-F	AGGATCTCAGACCGGTCGCACCTTCGGATATACTGT
PCV2(del191–)-R	AAAAAGATCTGCTAGCTTAAGAGGTTTGTAGCCTCAGCC
BFDV(del–50)-F	AGGATCTCAGACCGGTCGGCAGTTTCAGTTCAAGAT
BFDV(del191–)-R	AAAAAGATCTGCTAGCTTAGCCACCTTGCAGTGGAATCC

Supplementary Table S3. The amino acid sequence of mutant *Circoviridae* capsid proteins

**Capsid mutant**	**Amino acid sequence**
Chimera (BFDV(–101)/PCV2(101–))	MWGTSNCACAKFQIRRRYARPYRRRHIRRYRRRRRHFRRRRFTTNRVYTLRLTRQFQFKIQKQTTSVGNLIFNADYITFAL DDFLQAVPNPHALNFEDYRIKLVKVEFWPCSPITQGDRGVGSTAVILDDNFVTKATALTYDPYVNYSSRHTIPQPFSYHSR YFTPKPVLDSTIDYFQPNNKRTQLWLRLQTSRNVDHVGLGTAFENSIYDQDYNIRVTMYVQFREFNLKDPPLKP
Chimera (PCV2(–101)/BFDV(101–))	MTYPRRRYRRRRHRPRSHLGQILRRRPWLVHPRHRYRWRRKNGIFNTRLSRTFGYTVKATTVRTPSWAVDMMRFNIDDF VPPGGGTNKISIPFEYYRIRKAKMEMRPTGGHYTVQSNGFGHTAVIQDSRITKFKTTADQTQDPLAPFDGAKKWFVSRGFK RLLRPKPQITIEDLTTANQSAALWLNSARTGWIPLQGGPNSAGTKVRHYGIAFSFPQPEQTITYVTKLTLYVQFRQFAPNNP ST
BFDV(del–50)	RQFQFKIQKQTTSVGNLIFNADYITFALDDFLQAVPNPHALNFEDYRIKLAKMEMRPTGGHYTVQSNGFGHTAVIQDSRIT KFKTTADQTQDPLAPFDGAKKWFVSRGFKRLLRPKPQITIEDLTTANQSAALWLNSARTGWIPLQGGPNSAGTKVRHYGI AFSFPQPEQTITYVTKLTLYVQFRQFAPNNPST
BFDV(del191–)	MWGTSNCACAKFQIRRRYARPYRRRHIRRYRRRRRHFRRRRFTTNRVYTLRLTRQFQFKIQKQTTSVGNLIFNADYITFAL DDFLQAVPNPHALNFEDYRIKLAKMEMRPTGGHYTVQSNGFGHTAVIQDSRITKFKTTADQTQDPLAPFDGAKKWFVSR GFKRLLRPKPQITIEDLTTANQSAALWLNSARTGWIPLQGG
PCV2(del–50)	RTFGYTVKATTVRTPSWAVDMMRFNIDDFVPPGGGTNKISIPFEYYRIRKVKVEFWPCSPITQGDRGVGSTAVILDDNFVT KATALTYDPYVNYSSRHTIPQPFSYHSRYFTPKPVLDSTIDYFQPNNKRTQLWLRLQTSRNVDHVGLGTAFENSIYDQDYNI RVTMYVQFREFNLKDPPLKP
PCV2(del191–)	MTYPRRRYRRRRHRPRSHLGQILRRRPWLVHPRHRYRWRRKNGIFNTRLSRTFGYTVKATTVRTPSWAVDMMRFNIDDF VPPGGGTNKISIPFEYYRIRKVKVEFWPCSPITQGDRGVGSTAVILDDNFVTKATALTYDPYVNYSSRHTIPQPFSYHSRYFT PKPVLDSTIDYFQPNNKRTQLWLRLQTS

Supplementary Table S4. Genetic distance of *Circoviridae* capsid based on amino acid sequence The number of amino acids conserved per site between sequences of capsid proteins is shown.

No		1	2	3	4	5	6	7	8	9	10	11	12	13	14	15	16	17	18	19	20	21	22	23	24	25
1	ABU48444.1_CygCV	1.00																								
2	AAF74197.1_PiCV	0.19	1.00																							
3	QGM50354.1_PenCV	0.07	0.35	1.00																						
4	QGX08854.1_PCV4	0.16	0.27	0.17	1.00																					
5	ANO40512.1_PCV3	0.17	0.23	0.16	0.21	1.00																				
6	AAC59463.1_PCV2	0.17	0.25	0.19	0.44	0.25	1.00																			
7	NP_065679.1_PCV1	0.17	0.22	0.19	0.37	0.21	0.65	1.00																		
8	AAC69862.1_BFDV	0.23	0.40	0.43	0.20	0.21	0.25	0.24	1.00																	
9	ADD62476.1_HuACV1	0.13	0.20	0.19	0.19	0.16	0.19	0.18	0.20	1.00																
10	ABI54258.1_FiCV	0.27	0.42	0.38	0.23	0.21	0.25	0.23	0.40	0.21	1.00															
11	QVK11261.1_EquCV	0.17	0.21	0.18	0.36	0.22	0.53	0.56	0.24	0.17	0.22	1.00														
12	AAP69227.1_MDuCV	0.27	0.18	0.15	0.16	0.22	0.22	0.23	0.20	0.16	0.18	0.23	1.00													
13	UPW41432.1_DipV_4537	0.29	0.20	0.19	0.29	0.17	0.21	0.22	0.24	0.19	0.21	0.22	0.16	1.00												
14	AXQ04846.1_MosACV1	0.01	0.53	0.41	0.00	0.17	0.17	0.19	0.49	0.21	0.50	0.16	0.19	0.29	1.00											
15	ADD62466.1_ChimpACV	0.06	0.54	0.41	0.09	0.15	0.19	0.18	0.40	0.18	0.43	0.18	0.15	0.20	0.66	1.00										
16	CAD23544.1_CaCV	0.24	0.43	0.38	0.23	0.21	0.25	0.24	0.40	0.20	0.60	0.22	0.19	0.22	0.56	0.43	1.00									
17	YP_007697653.1_CanineCV	0.20	0.19	0.16	0.24	0.20	0.21	0.20	0.24	0.12	0.18	0.21	0.19	0.48	0.23	0.18	0.18	1.00								
18	QDZ59983.1_BWhaleCV	0.24	0.21	0.18	0.24	0.21	0.24	0.19	0.22	0.17	0.19	0.22	0.15	0.23	0.23	0.18	0.19	0.23	1.00							
19	ALG92530.1_BatACV4	0.17	0.33	0.14	0.29	0.24	0.30	0.27	0.21	0.19	0.24	0.27	0.23	0.17	0.01	0.14	0.23	0.14	0.21	1.00						
20	AIX11623.1_BatCyVPOAII	0.09	0.07	0.08	0.06	0.06	0.06	0.08	0.06	0.06	0.08	0.09	0.07	0.05	0.10	0.07	0.10	0.06	0.04	0.03	1.00					
21	AFK85002.1_ BatACV3	0.14	0.27	0.18	0.27	0.20	0.30	0.29	0.22	0.17	0.18	0.29	0.24	0.20	0.16	0.17	0.21	0.17	0.23	0.69	0.10	1.00				
22	AGL09952.1_ BatACV2	0.11	0.23	0.21	0.40	0.25	0.49	0.53	0.26	0.16	0.26	0.43	0.22	0.21	0.23	0.22	0.23	0.20	0.19	0.27	0.08	0.28	1.00			
23	AGL09970.1_BatACV1	0.17	0.19	0.15	0.30	0.22	0.36	0.33	0.21	0.16	0.21	0.32	0.22	0.17	0.11	0.15	0.18	0.19	0.21	0.30	0.08	0.27	0.32	1.00		
24	ARV76499.1_DuACyV1	0.11	0.14	0.13	0.23	0.11	0.16	0.15	0.16	0.15	0.14	0.14	0.12	0.14	0.16	0.13	0.15	0.15	0.15	0.19	0.05	0.13	0.15	0.15	1.00	
25	ADD62462.1_CyVc11	0.07	0.09	0.10	0.03	0.06	0.07	0.06	0.14	0.09	0.11	0.09	0.09	0.10	0.09	0.09	0.09	0.10	0.07	0.04	0.10	0.09	0.09	0.09	0.04	1.00

Supplementary Table S5. Multiple comparisons of the effect on pig TRIF-mediated IFN-β signaling between PCV1, PCV2, PCV3, and PCV4 capsid proteins.

	**PCV1**	**PCV2**	**PCV3**	**PCV4**
**PCV1**		**<0.0001**	**0.6957**	**<0.0001**
**PCV2**	**<0.0001**		**<0.0001**	**<0.0001**
**PCV3**	**0.6957**	**<0.0001**		**<0.0001**
**PCV4**	**<0.0001**	**<0.0001**	**<0.0001**	
