# Supplementary material for: Divergent Effects of Circoviridae Capsid Proteins on Type I Interferon Signaling
Source: Pathogens. 2025 Jan 13;14(1):68. doi: 10.3390/pathogens14010068 (PMC11768430; doi:10.3390/pathogens14010068)
Supplement: Supplementary file 1 [file pathogens-14-00068-s001.zip › 241215_Supplementary Figure_2nd_revise_AK5_AS Final.pdf]

Supplementary Figure S1

PCV2

cNLS Mapper Result

Predicted NLSs in query sequence
MTYPRRRYRRRRHRPRSHLGQILRRRPWLVHPRHRYRWRRKNGIFNTRLS 50<br>RTFGYTVKATTVRTPSWAVDMMRFNIDDFVPPGGGTNKISIPFEYYRIRK 100<br>VKVEFWPCSPITQGDRGVGSTAVILDDNFVTKATALTYDPYVNYSSRHTI 150<br>PQPFSYHSRYFTPKPVLDSTIDYFQPNNKRTQLWLRLQTSRNVDHVGLGT 200<br>AFENSIYDQDYNIRVTMYVQFREFNLKDPPLKP 233

Predicted monopartite NLS		
Pos.	Sequence	Score

Predicted bipartite NLS		
Pos.	Sequence	Score
5	RRRYRRRRHRPRSHLGQILRRRPWLVHPRHRYRW	5.2
5	RRRYRRRRHRPRSHLGQILRRRPWLVHPRHRYRW	5.2

BFDV

cNLS Mapper Result

Predicted NLSs in query sequence
MWGTSNCACAKFQIRRRYARPYRRRHIRRYRRRRRHFRRRRFTTNRVYTL 50<br>RLTRQFQFKIQKQTTSVGNLIFNADYITFALDDFLQAVPNPHALNFEDYR 100<br>IKLAKMEMRPTGGHYTVQSNGFGHTAVIQDSRITKFKTTADQTQDPLAPF 150<br>DGAKKWFVSRGFKRLLRPKPQITIEDLTTANQSAALWLNSARTGWIPLQG 200<br>GPNSAGTKVRHYGIAFSFPQPEQTITYVTKLTLYVQFRQFAPNNPST 247

Predicted monopartite NLS		
Pos.	Sequence	Score

Predicted bipartite NLS		
Pos.	Sequence	Score
208	KVRHYGIAFSFPQPEQTITYVTKLTLY	4.8

Supplementary Figure S2

Suppressing

PiCV
CanineCV
PCV1
PCV2
PCV3
BatACV1
BatACV2
EquCV
MosACV1

Enhancing

BFDV
ChimpACV
BatACV3
BatACV4 CaCV
DipV_4537
DuACyV1 FiCV

No effect

PenCV
BWhaleCV
BatCyVPOA Ⅱ
CyVc11
CygCV
MDuCV
HuACV1
PCV4

Association with pathogenicity:

Yes

No

Unknown

Supplementary Figure S3

Suppressing	Enhancing	No effect
PiCV	BFDV ChimpACV	PenCV
CanineCV	BWhaleCV	BatCyVPOA Ⅱ
PCV1	BatACV3 MDuCV	CaCV
PCV2	FiCV	CyVc11
PCV3		DipV_4537
PCV4		DuACyV1
BatACV1		HuACV1
BatACV2		
BatACV4		
CygCV		
EquCV		
MosACV1		

Association with pathogenicity:

Yes

No

Unknown
